# Benchmarking methods for measuring biosynthetic gene cluster similarity and determination of gene cluster families

**DOI:** 10.1093/bioinformatics/btaf636

**Published:** 2025-11-21

**Authors:** Abiodun S Oyedele, Allison S Walker

**Affiliations:** Department of Chemistry, Vanderbilt University, Nashville, TN 37240, United States; Department of Chemistry, Vanderbilt University, Nashville, TN 37240, United States; Department of Biological Sciences, Vanderbilt University, Nashville, TN 37235, United States; Department of Pathology, Microbiology, and Immunology, Vanderbilt University Medical Center, Nashville, TN 37232, United States

## Abstract

**Motivation:**

Natural products are often produced by a set of biosynthetic enzymes that are encoded by genes clustered together in the producer’s genome, referred to as a biosynthetic gene cluster (BGC). The ability to compare and cluster BGCs is essential for several applications, including predicting which bacteria will make a known product and assessing the potential diversity of natural products produced by a set of bacteria. There are multiple methods for comparing and clustering BGCs based on their similarity, but there has been a lack of investigation into how strongly BGC similarity relates to product structural similarity and how these methods perform relative to each other.

**Results:**

Using publicly available databases, we developed a benchmark dataset to assess how well different BGC similarity metrics correlate with the structural similarity of their products and how well these methods cluster BGCs. We found that all methods showed moderate correlation between BGC and structural similarity, with correlations improving for more similar BGCs and varying significantly by BGC biosynthetic class. Analysis of outliers revealed some outliers were due to mistakes or omissions in public datasets, while others represented deviation between BGC similarity and product structural similarity. All methods generally performed better on clustering metrics, with BiG-SCAPE performing the best after errors in the public datasets had been corrected.

**Availability and implementation:**

Scripts and data required to reproduce the results are available at https://github.com/aswalker-lab/BGC-clustering-benchmark and processed similarity, clusters, and scaffolds are also available at https://huggingface.co/datasets/allie-walker/BGC-clustering-benchmark. Code is also available at Zenodo: 10.5281/zenodo.17373546

## 1 Introduction

Natural products are an important source of therapeutics (Newman and Cragg 2020); however, in recent years, the rate of natural product discovery has slowed, in part due to the frequent rediscovery of known compounds (Atanasov *et al.* 2021). One approach that researchers have taken to overcome the challenge of rediscovery is to analyze the similarity of predicted biosynthetic gene clusters (BGCs) in a genome to BGCs that produce known products, to assess the likely novelty of the natural products produced by the associated organism (Gavriilidou *et al.* 2022). In other cases, researchers may be interested in discovering analogs of therapeutically promising known natural products that may have improved activity (Ancajas *et al.* 2024), for example, in the case of the tryptophan dimer class of natural product (Chang and Brady 2013, Chang *et al.* 2013, 2015). Both cases require the ability to bioinformatically compare two BGCs to determine how similar the structures of their products are likely to be. Beyond pairwise comparison, clustering of BGCs into BGC families allows researchers to assess the overall diversity of biosynthetic pathways and to assess the prevalence of specific BGC families across a set of genomes (Gavriilidou *et al.* 2022).

There have been multiple methods reported that enable comparison or clustering of BGCs. These include KnownClusterBlast, which is part of antiSMASH (Blin *et al.* 2023) for cluster comparison and BiG-SCAPE (Navarro-Muñoz *et al.* 2020), BiG-SLiCE (Kautsar *et al.* 2021), clust-o-matic (Gavriilidou *et al.* 2022), and lsaBGC (Salamzade *et al.* 2023) for both BGC comparison and clustering. However, there is a lack of benchmarks to assess which methods will perform best for a user’s specific application. There are also other methods that allow for the comparison of BGCs, such as MultiGeneBLAST (Medema *et al.* 2013), CAGECAT (van den Belt *et al.* 2023), and GATOR-GC (Cediel-Becerra *et al.* 2025) that do not automatically cluster BGCs or have quantitative pairwise BGC similarity scores, making them more difficult to benchmark. Therefore, we chose to focus only on methods with pairwise BGC similarity scores or that cluster BGCs into BGC families.

Researchers may have differing goals when comparing BGCs, and therefore the benchmarks proposed here may not be relevant to all applications. We have chosen to focus on benchmarking how similarity scores for BGCs compare to the similarity of the products they produce for the purpose of dereplication of known compounds and targeted discovery of analogs of a product of interest. However, researchers might be interested in comparing and clustering BGCs based on their evolutionary relationship. While these problems are related, they are not the same, for example, there are multiple cases of convergent evolution of BGCs, where BGCs with distinct evolutionary history produce similar products (Fischbach 2009, Chevrette and Currie 2019, Parkinson *et al.* 2019, Ju and Nair 2022).

Another challenge facing the benchmarking task is that unsupervised clustering often has no ground truth, and therefore, the quality of the clusters depends on the specific application the user would like to use the clusters for. One user may want to cluster BGCs in such a way that all members of a cluster produce identical products, while another might want a cluster to produce compounds that share a scaffold. Another challenge is that BGCs and their products may not form clean clusters. For example, BGC 1 could produce compound 1 that has one substructure in common with compound 2 produced by BGC 2 and another substructure in common with compound 3 produced by BGC 3, a phenomenon which has been observed in natural products and can occur through new combinations of subclusters (Medema *et al.* 2014). In this case, it is not clear if BGC 1 should be clustered with BGC 2 or BGC 3, if it should be in its own cluster, or if it should connect the BGC 2 and BGC 3 clusters into one larger cluster. We have chosen to assess clustering based on established unsupervised clustering metrics and by comparing how similar BGC clusters are to compound clusters. We therefore encourage readers to carefully consider which metrics are most relevant to their application when deciding which method to use.

## 2 Materials and methods

### 2.1 Assembly of benchmark set

We assembled our benchmark set from NPAtlas which was downloaded NPAtlas on 28 February 2024, version 2023_06 (van Santen *et al.* 2019) and the links to MIBiG BGC identifiers were used. Pairwise Tanimoto similarity for all natural products in NPAtlas was calculated using RDKit’s Fingerprint Generator with a bit size of 8192, which is larger than most commonly used fingerprints but was chosen due to the larger size and complexity of natural products relative to synthetic compounds, and RDKit’s Tanimoto similarity calculation. If BGCs produced multiple products, then the highest pairwise Tanimoto score across all combinations of products from each BGC was used. Product clusters were created using RDKit’s Butina clustering method applied to the RDKit fingerprints, thresholds of 0.2 and 0.3 were chosen based on Silhouette scores, calculated using scikit-learn’s silhouette_score metric (Table 1, available as supplementary data at *Bioinformatics* online). Bemis-Murcko scaffolds were generated by first removing the molecule’s stereochemistry in RDKit and then applying RDKit’s MurckoScaffold. The resulting scaffold was cleaned by replacing the substructure “[$([D1]=[*])]” in SMARTS with “[*]” using RDKit’s ReplaceSubstructure method. Through the course of this work, we identified several incorrect BGC-product pairs or missing products for some BGCs by analyzing pairs with extreme discrepancies in BGC and structural similarity followed by manual analysis of the BGC. To create a final benchmark set, we removed any incorrect BGC-product pairs and added BGC-product pairs for missing products. BGC files were downloaded from the MIBiG database (Zdouc *et al.* 2025).

**Table 1.** Correlation between BGC similarity score (knownclusterblast, BiG-SCAPE, and lsaBGC) and distance score (BiG-SLICE and clust-o-matic) and product structural similarity.^a^

**Method**	Spearman’s rho
**Knownclusterblast (similarity)**	**0.47**
BiG-SCAPE 0.3 cutoff (similarity)	0.26
BiG-SCAPE 0.5 cutoff (similarity)	0.40
BiG-SCAPE 1 cutoff (similarity)	0.26
BiG-SLICE (distance)	−0.19
Clust-o-matic (distance)	−0.28
Clust-o-matic with distances greater than 0.5 excluded (distance)	−0.19
lsaBGC (similarity)	0.19
lsaBGC with threshold of 50 (similarity)	0.31

a The best-scoring method, knownclusterblast, is shown in bold.

### 2.2 Running antiSMASH and extracting KnownClusterBlast values

AntiSMASH 7.1.0 (Blin *et al.* 2023) was run on all GenBank files. The KnownClusterBlast scores were extracted from the output knowncluster blast svg file, ending with _all which is found in the svg directory of antiSMASH output. If an input file produced two predicted antiSMASH clusters, which happened in only six cases for which there was an NPAtlas link, only one was used in subsequent similarity score and clustering metric calculations. AntiSMASH did not recognize some of the clusters as BGCs, these were therefore excluded from the KnownClusterBlast, BiG-SCAPE, BiG-SLiCE, and lsaBGC analysis, all of which rely on antiSMASH-annotated BGCs.

### 2.3 Running BiG-SCAPE

BiG-SCAPE version 1.1.2 (Navarro-Muñoz *et al.* 2020) was run on the output from antiSMASH 7 using cutoffs of 0.3, 0.5, and 1.0. BiG-SCAPE similarity scores were extracted from the BiG-SCAPE outputs, specifically the file network_files/<DATE_OF_RUN>_hybrids_glocal/mix/mix_c<THRESHOLD>.network found within the BiG-SCAPE output directory, where <DATE_OF_RUN> is a label applied by BiG-SCAPE corresponding to when the analysis was run and <THRESHOLD> is the threshold BiG-SCAPE was run with. These similarity scores represent the squared similarity.

### 2.4 Running BiG-SLiCE

BiG-SLiCE version 2.0.0 (Kautsar *et al.* 2021) was run on the antiSMASH 7 output with a threshold of 0.4, with the –export_tsv option to output cluster information which was then used in determining cluster quality.

### 2.5 Running clust-o-matic

Clust-o-matic (Gavriilidou *et al.* 2022) was run with a 0.5 cutoff on a concatenated fasta file of annotated proteins from the BGCs.

### 2.6 Running lsaBGC

lsaBGC is designed to run on whole genomes; therefore, we had to make some modifications to the pipeline to run it on individual BGCs. We treated the MIBiG sequences as genomic sequences and ran antiSMASH 7 on these individual clusters to generate antiSMASH output. We excluded fungal and plant BGCs from the lsaBGC analysis since it was developed for prokaryotic sequences. Because lsaBGC limits the number of genomes used for initial clustering of BGCs, we had to choose a subset of the MIBiG BGCs to be primary and others to be secondary. We first specified any BGCs not recognized by antiSMASH as secondary. We then identified BGCs that produce the same compound or congeners and moved one of the copies to a list of secondary clusters. After this analysis, we still needed to remove more BGCs from the primary list; we selected BGCs with no KnownClusterBlast hits in MIBiG as these are likely to be singletons. A few BGCs also led to errors when running lsaBGC and these were removed as well. A list of clusters designated primary and secondary is available in Table 2, available as supplementary data at *Bioinformatics* online. We then ran lsaBGC version 1.52 using the steps and parameters recommended on the creators’ wiki page (https://github.com/Kalan-Lab/lsaBGC/wiki).

**Table 2.** Spearman’s correlation for different natural product classes and distance measurement methods.^a^

Including Hybrid Clusters							
	PK	NRP	alkaloid	RiPP	Saccharide	Terpene	Other
Knownclusterblast	**0.43** (3026)	**0.49** (1975)	0.29 (49)	0.29 (165)	0.28 (140)	**0.62** (89)	**0.60** (91)
BiG-SCAPE 1.0 cutoff	0.21 (199 001)	0.38 (151 928)	0.27 (384)	0.49 (1957)	**0.49** (1729)	0.15 (1030)	0.20 (2265)
BiG-SLiCE	−0.11 (403 225)	−0.23 (305 809)	−0.15 (1296)	−0.13 (9025)	−0.49 (3844)	−0.37 (3600)	−0.28 (11 449)
Clust-o-matic	−0.23 (396 900)	−0.46 (298 116)	−0.22 (1296)	−0.29 (8836)	−0.40 (3844)	−0.21 (3364)	−0.25 (10 816)
lsaBGC	0.21 (43 956)	0.13 (28 680)	**0.77** (45)	**0.77** (28)	0.47 (630)	−0.02 (21)	0.42 (231)
No Hybrid Clusters							
Knownclusterblast	**0.37** (1407)	0.43 (950)	0.26 (37)	0.30 (143)	0.44 (47)	**0.48** (39)	**0.58** (69)
BiG-SCAPE 1.0 cutoff	0.30 (64 207)	0.51 (46 317)	0.38 (172)	0.51 (1816)	0.57 (170)	0.23 (464)	0.21 (1600)
BiG-SLiCE	−0.21 (131.044)	−0.38 (93 636)	−0.25 (529)	−0.14 (8281)	**−0.58** (400)	−0.42 (1764)	−0.26 (8836)
Clust-o-matic	−0.29 (128 881)	**−0.56** (90 000)	−0.35 (529)	−0.30 (8100)	−0.50 (400)	−0.18 (1681)	−0.24 (8281)
lsaBGC	0.34 (14 878)	0.15 (8646)	**0.80** (36)	**0.77** (28)	0.53 (66)	−0.26 (10)	0.34 (153)

a The best score for a class is bold and the best score for a method is underlined. The upper part of the table includes hybrid clusters (those with more than one biosynthetic class) while the bottom part of the table excludes them. Correlations are rounded to the nearest hundredth, but further decimals were used to break ties when determining the best score. The number of pairs in the analysis for each method is shown in parentheses.

### 2.7 Calculation of benchmark metrics

Correlation between BGCs and Tanimoto similarity using Spearman’s rho calculated with a Python script using scipy. Clustering metrics were calculated using scikit-learn’s metrics.

## 3 Results and discussion

### 3.1 Gene cluster similarity and structural similarity correlation

We first set out to determine how well different BGC similarity scoring metrics correlate with structure similarity. To do this, we assembled a dataset of BGC-natural product pairs using the linkages between the NPAtlas (van Santen *et al.* 2019) and MIBiG (Terlouw *et al.* 2023) datasets. We then determined similarity or distance scores using antiSMASH’s KnownClusterBlast (Blin *et al.* 2013), BiG-SCAPE (Navarro-Muñoz *et al.* 2020), BiG-SLiCE (Kautsar *et al.* 2021), clust-o-matic (Gavriilidou *et al.* 2022), and lsaBGC (Salamzade *et al.* 2023). Some of these methods do not determine similarity for BGCs that do not meet a specific threshold. For those cases, we only included BGCs for which a score was calculated in subsequent analysis. BiG-SCAPE allows the user to set a cutoff threshold, so we tried running BiG-SCAPE with multiple thresholds, specifically, the default threshold (0.3), the maximum possible distance (1.0), and an intermediate value (0.5), to see how that affected performance. We then determined the correlation between the BGC similarity or distance scores and the molecular similarity, as determined by the Tanimoto coefficient (Bajusz *et al.* 2015) (Table 1 and Fig. 1, available as supplementary data at *Bioinformatics* online). For cases where there were multiple products for one BGC in NPAtlas, we used the maximum Tanimoto coefficient among all pairs of products between the two BGCs.

All methods showed statistically significant correlation as measured by Spearman’s rho (*P* < .0005 for all methods) and had the expected sign with similarity scores showing a positive correlation and distances showing a negative correlation. Despite their significance, the magnitude of all correlations for all methods indicated weak to moderate correlations (ranging from 0.19 to 0.47). The best performing method was KnownClusterBlast with a Spearman’s rho of 0.47. It is important to note that KnownClusterBlast does not calculate a score for pairs of BGCs with homology below a certain threshold. Therefore, these BGCs are not included in the analysis. This may give the method an unfair advantage, as the performance of all methods seems to degrade for very distantly related BGCs. For example, BiG-SCAPE was very sensitive to the choice of cutoff (with a rho of 0.40 at a 0.5 cutoff versus a rho of 0.26 for cutoffs of 0.3 or 1). Clust-o-matic and lsaBGC were also sensitive to the cutoff for scores included when calculating correlation. Clust-o-matic had a rho of −0.28 with no cutoff and a rho of −0.19 using the recommended clustering threshold of 0.5. lsaBGC improved from a rho of 0.19–0.31 when it was thresholded to a score of 50, the default cluster threshold. It is important to note that the recommended defaults for all of these methods are the threshold at which two BGCs are clustered into a Gene Cluster Family (GCF), not the ideal criteria for trusting a similarity or distance score. As these methods were primarily developed for clustering and not pairwise comparison, there is no recommended threshold specifically for the type of correlation analysis we are performing. If readers are interested in a method’s ability to compare both distantly and closely related BGCs, they should refer to the unthresholded scores when choosing a method, while the thresholded values are better for assessing whether a method will perform well on more closely related BGCs. Visual inspection of the plots of BGC similarity versus Tanimoto similarity also reveals that performance is very poor for more distantly related compounds and BGCs, which could explain why KnownClusterBlast performs better than other methods (Fig. 1, available as supplementary data at *Bioinformatics* online). It is also important to note that while BiG-SLiCE performed worse than other methods, it is significantly faster and capable of processing many more BGCs than BiG-SCAPE, and therefore BiG-SLiCE may be the only choice when dealing with large datasets (Kautsar *et al.* 2021).

### 3.2 Examination of BGC similarity outliers reveals database errors and omissions, as well as BGCs where KnownClusterBlast does not perform well at predicting structural similarity

Visual inspection of the plots of BGC similarity scores versus structural similarity scores revealed that all methods have extreme outliers, where the Tanimoto similarity is near one, but the BGC similarity is near zero or vice - versa. We further investigated the 10 top outliers in each direction for the method with the highest correlation, KnownClusterBlast, defined as the difference between the KnownClusterBlast score divided by 100 and the Tanimoto similarity score. KnownClusterBlast scores are asymmetric, so when removing duplicates where the order of the pairing was switched, we only had nine pairs per category. Details on these pairs are summarized in Tables 3 and 4, available as supplementary data at *Bioinformatics* online and pairs are visualized by KnownClusterBlast or clinker in Figs 2–5, available as supplementary data at *Bioinformatics* online).

**Table 3.** Silhouette clustering scores.^a^

	Silhouette—Method Score	Silhouette—Structure Tanimoto
BiG-SCAPE 0.3 cutoff	**0.71**	**0.56**
BiG-SCAPE 0.5 cutoff	0.50	0.36
BiG-SCAPE 1.0 cutoff	0.09	−0.04
BiG-SLiCE	0.09	0.04
Clust-o-matic 0.5 cutoff	0.14	0.10
lsaBGC	0.01	−0.07

a Best scores are shown in bold.

**Table 4.** Supervised clustering metrics.^a^

	Adjusted rand	Adjusted mutual information	V-measure	Fowlkes Mallow
Butina threshold = 0.2				
BiG-SCAPE 0.3 cutoff	0.28	0.35	**0.97**	0.32
BiG-SCAPE 0.5 cutoff	0.36	0.44	0.96	0.37
BiG-SCAPE 1.0 cutoff	0.13	0.24	0.84	0.21
BiG-SLiCE	0.37	0.44	0.96	0.38
Clust-o-matic 0.5 cutoff	**0.42**	**0.48**	**0.97**	**0.45**
lsaBGC	0.20	0.26	0.94	0.23
Butina threshold = 0.3				
BiG-SCAPE 0.3 cutoff	0.17	0.29	0.95	0.25
BiG-SCAPE 0.5 cutoff	**0.31**	**0.43**	0.95	0.31
BiG-SCAPE 1.0 cutoff	0.15	0.29	0.83	0.19
BiG-SLiCE	0.26	0.40	0.95	0.30
Clust-o-matic 0.5 cutoff	0.26	0.41	**0.96**	**0.34**
lsaBGC	0.23	0.30	0.93	0.23

a Best scores are shown in bold.

We first examined these BGCs to confirm that these differences were not due to errors in the MIBiG database. We found several of the discrepancies were likely due to truncated or split BGCs in MIBiG. The cluster for urdamycin (BGC0000277) appears to be truncated as there are no polyketide biosynthetic genes present in the cluster, including urdA, which is one of the polyketide synthases (PKS) genes needed for urdamycin biosynthesis.(Faust *et al.* 2000, von Mulert *et al.* 2004) This truncation leads to low BGC similarity with a number of BGCs that have similar polyketide cores (Table 3 and Figs 2 and 3, available as supplementary data at *Bioinformatics* online). We also found that two clusters, each reported to code for frankobactin (BGC0002409 and BGC0002410), are likely the same biosynthetic pathway that were split into two clusters in the genome or not assembled correctly in the original genome assembly. The original paper reports the cluster as “bipartite” and there are fewer nonribosomal peptide synthetases (NRPS) modules in each of the individual clusters compared to the original paper that reported the BGC (Mohr *et al.* 2021). In another example, the ochortoxin A BGC (BGC0002608) had an unexpectedly high KnownClusterBlast score of 100% with the BGC for chaetoviridin and 11-epichaetomugilin A (BGC0001405), this was because BGC0002608 contained only a single annotated gene which had homology to a gene in BGC0001405. BGC0002608 is likely either truncated or contains ORFs that were not properly annotated in the BGC region, as the original paper that reported this BGC described five ORFs (Gil-Serna *et al.* 2018). The gene cluster BGC0002192 is also likely truncated as it has only a single gene when the BGC described in the corresponding paper has six genes (Itoh *et al.* 2018), leading to too high of a similarity to BGC0000098. Another MIBiG error was found by comparing the BGCs for the svetamycins (BGC0002426) and BE-24566B (BGC0001512) which had a KnownClusterBlast score of 100% despite the structures of the products being from different biosynthetic classes, NRPS and PKS, respectively. Further examination of BGC0002426 revealed that it has Type-2 PKS and indole biosynthesis genes without any NRPS biosynthesis genes and that all genes are named with the prefix bab. Therefore, this cluster is likely the bab supercluster, which is reported to produce borregomycin and antrabenzoxocinone, indocarbazole and aromatic polyketides, respectively. This mistake was likely made because the svetamycins are produced by a different BGC in the same strain and the BGCs were reported in the same publication (Morshed *et al.* 2021). Overall, we caught six BGCs that were either incorrect or truncated in the MIBiG database. As of this writing, all of these BGCs are indicated to be of “questionable” quality and “unknown” completeness in MIBiG version 4.0 (Zdouc *et al.* 2025). However, this is the classification for many of the BGCs in MIBiG and so these labels alone do not indicate to users that these BGCs should be treated with any higher skepticism than other BGCs we did not find issues with. We intend to inform the managers of the MIBiG database of the errors upon publication of this manuscript, so these errors may not remain after publication.

In some cases, with low Tanimoto scores and high KnownClusterBlast scores, the discrepancies were caused by incomplete lists of products of BGCs, especially when comparing across the MIBiG and NPAtlas databases. BGC0002295 and BGC0000375 are completely homologous based on KnownClusterBlast and clinker (Figs 4 and 5, available as supplementary data at *Bioinformatics* online), but BGC0002295 was reported to produce both minimycin and indigoidine (Kong *et al.* 2019), whereas only minimycin is reported as a product in NPAtlas. BGC0000375 has only been reported to produce indigoidine (Yu *et al.* 2013). It is possible that this BGC also produces both products but that minimycin was missed during the initial study or that these BGCs produce different products due to slight differences in the sequence of minA and the corresponding indC, which is the gene responsible for controlling which product is produced (Kong *et al.* 2019). Regardless, if indigoine had also been included as a product of BGC0002295 in NPAtlas, these BGCs would have correctly received a Tanimoto score of 1, since our method takes the highest possible Tanimoto score across all pairs. Another similar example is the BGC pair, BGC0001433 and BGC0001296, which produce the argimycins and streptazone E, respectively. These products are structurally similar and accordingly the BGCs are also similar but NPAtlas only lists nigrifactin, an isolatable biosynthetic intermediate in the argimycin biosynthetic pathway (Ye *et al.* 2017), for BGC0001433 (Table 3 and Figs 4 and 5, available as supplementary data at *Bioinformatics* online). Nigrifactin is also an intermediate in the streptazone E pathway that has been observed in extracts from the producing strain (Ohno *et al.* 2015) but it is not reported as a product of BGC0001296 in either MIBiG or NPAtlas. This discrepancy highlights the importance of having a complete accounting of products in natural product databases, which is challenging given that it is easy to miss products of a BGC during the isolation process. It also raises the question of whether intermediates produced in large enough quantities to be characterized, such as nigrifactin, should be considered as products of the BGC for purposes of comparing the structural similarities of products. Like the issues with the MIBiG errors, we intend to report the missing product structures to the maintainers of NPAtlas and therefore, these errors may be corrected and no longer apply after publication of this work.

However, not all discrepancies are due to errors or omissions in MIBiG and NPAtlas. In two of the nine BGCs with low KnownClusterBlast scores and high Tanimoto coefficients, one of the BGCs had additional genes that are usually annotated as playing a role in transport, regulation, or as accessories to other biosynthetic genes and occasionally included core biosynthetic genes that were missing from the other cluster (Table 3 and Figs 2 and 3, available as supplementary data at *Bioinformatics* online). In these cases, it is possible that the smaller BGC is incomplete or that the larger BGC contains genes that are not part of the BGC, but there was insufficient evidence in the literature to support this conclusion definitively. Regardless, identification of BGC boundaries is a challenging problem (Almeida *et al.* 2022, Salamzade *et al.* 2023, Nuhamunada *et al.* 2024, Cediel-Becerra *et al.* 2025) and methods for clustering BGCs in a manner that is predictive of natural product structure should be designed to be robust to the inclusion of non-biosynthetic genes that are not truly part of the cluster. Another example that further highlights this issue is the pair of BGCs, BGC0002078 and BGC0002079. These BGCs contain the same number of genes in their MIBiG entries, and analysis with clinker shows that the clusters are fully homologous (Fig. 2, available as supplementary data at *Bioinformatics* online), but when the BGCs are processed by antiSMASH to run KnownClusterBlast, they are split into two BGCs, resulting in a lower KnownClusterBlast relative to the MIBiG entry they are compared to (Fig. 3, available as supplementary data at *Bioinformatics* online).

For BGCs with high KnownClusterBlast scores and low Tanimoto coefficients, in all cases where there were no database errors, the high KnownClusterBlast score was due to one of the BGCs having significantly more biosynthetic genes than the other (Table 4 and Figs 4 and 5, available as supplementary data at *Bioinformatics* online). When antiSMASH has a larger BGC as the query and there is a smaller MiBIG cluster that only contains homologs to genes contained in the larger cluster, the KnownClusterBlast score will be reported as 100% (Figs 2 and 4, available as supplementary data at *Bioinformatics* online). This leads to misleading similarity scores because if the larger query BGC contains many additional biosynthetic genes, then its product is unlikely to have a similar structure to that of the smaller cluster. This problem is further compounded by the fact that some NRPS and PKS genes have similar enough sequences that KnownClusterBlast considers them to be homologs even though they have low sequence identity and different numbers of modules. For example, in the BGCs for Le-pyrrolopyrazines and gamexpeptide C, one has three NRPS genes with three, two, and one modules, while the other has one NRPS gene with five modules, but the genes have sequence identities as high as 46.19% as measured by BLAST using the gamexpeptide C NRPS as the query, which could explain why KnownClusterBlast marks the genes as homologs (Fig. 5, available as supplementary data at *Bioinformatics* online). The issue with KnownClusterBlast caused by BGCs with few genes could potentially be mitigated by reversing the query and subject BGCs and recalculating the score, as this leads to a more reasonable score in these cases. For example, when looking at the pair BGC0001703 and BGC0002480, the KnownClusterBlast score is 100% using BGC0001703 as the query but 31% using BGC0002480 as the query, which is more in line with the Tanimoto score of 0.09.

Despite these issues with KnownClusterBlast, it was still the highest performing method, meaning that it is likely that the other methods also have similar drawbacks. Namely, that similarity thresholds for homology may need to be varied for different types of genes or domains in order to be more predictive of product structural similarity and that some genes such as regulatory genes and transporters do not play a role in product structure and therefore, if these genes differ they will lead to cluster similarity scores that do not align with product structural similarity. In addition, despite the lack of accuracy of KnownClusterBlast in comparing BGCs in terms of their products’ similarity, it was still helpful for flagging multiple errors or omissions in the MIBiG and NPAtlas databases, highlighting how the effort of benchmarking BGC comparison methods can reveal these issues. These errors have persisted through multiple versions of MIBiG including the most recent that involved a massive community curation effort involving 267 contributors (Zdouc *et al.* 2025). An improved method for comparing BGCs would likely make it even easier to catch these errors and could be an essential tool for future updates to databases such as MIBiG.

### 3.3 Strength of correlation varies with natural product class

We next investigated whether the strength of correlation across the different methods varied with different natural product classes (e.g. nonribosomal peptides, polyketides, etc). We expected this to be the case because some classes are very rich in terms of how much information the BGC provides about the structure of the natural product. For example, the monomers used by NRPS and PKS can be predicted from the sequence of the domains in the NRPS and PKS (Yadav *et al.* 2003, Chevrette *et al.* 2017, Helfrich *et al.* 2019, Sokolova *et al.* 2024, Adduri *et al.* 2025, Terlouw *et al.* 2025). The modularity of NRPS and PKS may be advantageous for predicting structure because the connection between a module and what portion of the molecule it biosynthesizes is often clearer than for non-modular biosynthetic pathways. However, they also tend to have repetitive sequences (Gomez-Escribano *et al.* 2016), which lead to a challenge for methods like KnownClusterBlast which act on the protein rather than the domain level. This method will often recognize two NRPS or two PKS BGCs as similar even if they have differences in the total number of modules, for example, the comparison of gamexpeptide C and Le-pyrrolopyrazines shown in the first example in Fig. 5, available as supplementary data at *Bioinformatics* online. In addition, these BGCs often have distinctive tailoring enzymes that install different types of modifications on the product’s scaffold. In some cases, such as enterocin (Piel *et al.* 2000), a single tailoring enzyme may have a large effect on the structure, weakening the relationship between overall BGC similarity and product similarity. Conversely, terpene BGCs are relatively information-poor—generally all terpene BGCs contain terpene synthases and cyclases as well as P450s that are responsible for oxidizing the scaffold. In addition to the lower diversity of tailoring enzymes compared to NRPS and PKS pathways, the relationship between the sequence of proteins involved in terpene biosynthesis and the product’s structure is less clear compared to NRPS and PKS domains (Leferink and Scrutton 2022). We anticipate that the class of ribosomally synthesized and posttranslationally modified peptides (RiPPs) will behave differently than other BGCs. This is because the chemical structure of RiPPs is largely encoded by the sequence of the precursor peptide, and most of the modifying enzymes make alterations that are unlikely to significantly affect the Tanimoto coefficients of the final RiPP product.

As expected, accuracy varied significantly by BGC class (Table 2 and Figs 6–10, available as supplementary data at *Bioinformatics* online). Unexpectedly, the various methods differed in which class performed best, and the observed trends did not align with our previous expectations. For instance, when hybrid natural products are included, terpenes emerge as the top class for KnownClusterBlast analysis, whereas PKS and NRPS exhibit lower correlations. Some methods that perform very poorly on certain classes perform the best on others. For example, lsaBGC had one of the worst overall performances, but clearly had the strongest performance for alkaloids and RiPPs, regardless of whether hybrid clusters were excluded. We performed the correlations both including hybrid clusters, which are clusters with more than one class of natural product, and excluding hybrid clusters. Correlations when including hybrid clusters were more accurate for KnownClusterBlast (5/7 classes), but generally more accurate when excluding hybrids for most of the other methods. However, these differences are relatively small.

We found it unusual that lsaBGC performed so much better in the alkaloid and RiPP classes relative to the other categories. We found this partly an artifact of our filtering of BGCs included in lsaBGC, when these BGCs were also excluded from the BiG-SCAPE analysis correlation rose to 0.38 for alkaloids and 0.62 for RiPPs, with hybrids included. Increased correlation was also seen for all other methods except KnownClusterBlast for alkaloids (Table 5, available as supplementary data at *Bioinformatics* online), but, lsaBGC remained the best method for alkaloids and RiPPs. The remaining improvement of lsaBGC for these categories can be accounted for by looking at what pairs lsaBGC determined a score for. When we only included the same pairs in our analysis of BiG-SCAPE, we found that correlation improved to 0.82 for alkaloids and 0.80 for RiPPs including hybrids, which slightly exceeds the correlation of lsaBGC. Correlations for alkaloids and RiPPs for all other methods also improved when limiting analysis to the pairs used by lsaBGC (Fig. 6, available as supplementary data at *Bioinformatics* online). These results suggest that the improvement in lsaBGC for these categories is due to our filtering procedure used to select which BGCs would be analyzed by IsaBGC rather than an intrinsic property of lsaBGC.

**Table 5.** Molecular scaffolds and GCFs.^a^

Clustering Method	Normalized Scaffold per GCF	GCFs per Scaffold
BiG-SCAPE 0.3 cutoff	**1.04**	1.22
BiG-SCAPE 0.5 cutoff	1.17	1.18
BiG-SCAPE 1.0 cutoff	3.34	1.14
BiG-SLiCE	1.04	1.24
Clust-o-matic 0.5 cutoff	1.07	1.19
lsaBGC	1.32	**1.11**

a Best scores are shown in bold.

### 3.4 Analysis of cluster quality

We next investigated the quality of clusters, which we will also refer to as GCFs, produced by the different methods using both the Silhouette scores for the BGC similarities and Tanimoto similarities of the corresponding products (Table 3). In both cases, BiG-SCAPE with a 0.3 cutoff performed the best while BiG-SLiCE and lsaBGC performed the worst, with Silhouette scores near zero. To further investigate cluster quality, we also clustered the compounds themselves using the Butina clustering method (Butina 1999), an established method that clusters molecules based on their Tanimoto similarity scores, at two different thresholds, 0.2 and 0.3. We compared cluster labels to BGC clusters using adjusted Rand, adjusted mutual information, V-measure, and Fowlkes Mallow. At the Butina threshold of 0.2, clust-o-matic performed the best or was tied on all metrics (Table 4). At the Butina threshold of 0.3 BiG-SCAPE with a cutoff of 0.5 performed best according to adjusted Rand and adjusted mutual information, while clust-o-matic had the best performance as measured by the other metrics. As with the correlation results, it is important to note that while BiG-SLiCE did not perform as well as many of the other methods, it is more scalable than the others (Kautsar *et al.* 2021).

Finally, we looked at how well different clustering methods captured similar scaffolds. To do this, we first determined the Bemis-Murko scaffolds, which represent molecules as their cyclic components and the atoms in these components (Bemis and Murcko 1996) for each BGC’s products. We then counted all scaffolds in each GCF and normalized them to the maximum number of scaffolds produced by a single BGC within the GCF. By this metric, a score of one indicates that all scaffolds produced by BGCs in the GCF are also produced by at least one other BGC in the GCF, while a score higher than one indicates that some BGCs in the GCF produce scaffolds that other BGCs do not. We then generated histograms (Fig. 11, available as supplementary data at *Bioinformatics* online) and averaged across all GCFs (Table 5). All methods, except BiG-SCAPE with a high threshold (1.0), performed well with averages only slightly greater than one. We also examined how many scaffolds were shared between different GCFs. To do this, we determined the average number of GCFs in which each scaffold occurred (Fig. 12, available as supplementary data at *Bioinformatics* online, Table 5). All methods also performed well and similarly in this case with the average number of GCFs per scaffold being close to one.

### 3.5 Benchmarking script for analysis of new similarity or clustering methods

It is likely that new methods for determining BGC similarity or for clustering BGCs into GCFs will be published in the future. To enable easy benchmarking of existing methods against these new methods, we have developed Python scripts to output the various metrics described above. For this benchmarking, we removed the BGCs we identified above as being incorrect and added missing products. These changes did not lead to significant differences in the correlations reported above (Tables 7–11, available as supplementary data at *Bioinformatics* online). Due to this small change after removing these BGCs, we also believe that if there are additional incorrect BGC-product pairs in our dataset, they will have only a small effect on benchmarking results. For clustering benchmarks, most metrics did not change significantly, except in the case of BiG-SCAPE with a 0.3 or 0.5 cutoff, which showed significant improvement in the adjusted Rand, adjusted mutual information, and Fowlkes mallow clustering metrics on the corrected dataset, making BiG-SCAPE with a 0.3 cutoff the best-scoring method on these three metrics in the corrected dataset. Benchmarking results for all methods with the new dataset are reported in Tables 5–9, available as supplementary data at *Bioinformatics* online. This benchmarking script and associated instructions have been made available on Github: https://github.com/aswalker-lab/BGC-clustering-benchmark.

The existing methods we analyzed in this work are all based on comparison of protein or domain sequences. This type of approach may not be able to fully capture the complexities of biosynthesis. One potential solution is to use foundation models, which are deep learning models trained on large amounts of unlabeled data that can then be fine-tuned for specific supervised applications on a smaller quantity of labeled data (Guo *et al.* 2025). Genomic language models (Nguyen *et al.* 2024, Benegas *et al.* 2025, Consens *et al.* 2025, Kang *et al.* 2025, Zhou *et al.* 2025) are foundation models that process genomic sequences, and these could possibly be applied to BGC-related tasks such as determining whether two BGCs will produce similar products or to cluster BGCs. In addition, there are several self-supervised BGC-specific deep learning models designed that may be suitable as foundation models (Rios-Martinez *et al.* 2023, Lai *et al.* 2025, Liu *et al.* 2025) for these tasks. So far, these models have been applied to identify BGCs and sometimes to link BGCs to possible products or broad product classes. In theory, foundation models can learn more complex relationships between BGC sequence and product structure, but their accuracy will depend on the quantity and quality of data available for fine-tuning. The benchmark reported here will enable comparisons between these emerging models and sequence similarity-based methods.

## 4 Conclusion

We developed a benchmarking dataset and associated metrics to assess how well various methods compare and cluster BGCs relative to the structural similarity of their products. We found that all methods had at best a moderate correlation between BGC similarity and product similarity. This indicates that overall BGC genetic content may not be that predictive of its product structure and that new methods are needed that rely more strongly on those genes that are predictive of product structure. Despite this, we found that all methods performed better at clustering with BiG-SCAPE performing the best, although there is still room for improvement, especially according to the Adjusted Rand, Mutual Information, and Fowlkes Mallow metrics.

It is also important to note that the public databases used in this work are incomplete and may still contain errors, even after we corrected some errors we found during this work. We expect that any errors in the database will have minimal impact on the metrics, as there are 1438 clusters in the final dataset and errors are relatively rare. The databases we used to build our benchmark contain a large diversity of natural products from different structural classes and biological sources, but there are some classes that are poorly represented and that are characterized as “other” in the benchmarks described in our results. It is possible that comparison and clustering methods will perform differently than expected from the metrics we have calculated when applied to BGCs that are not from one of the major classes. Overall, this work will enable improvements in methods for comparing and clustering BGCs, support evaluation of emerging deep learning foundation models, and provide insight into the relationship between BGCs and their products.

## Supplementary Material

btaf636_Supplementary_Data

## Data Availability

The data required to reproduce the results are available from Github at https://github.com/aswalker-lab/BGC-clustering-benchmark and processed similarity, clusters, and scaffolds are also available from Hugging Face at https://huggingface.co/datasets/allie-walker/BGC-clustering-benchmark
