# Supplementary material for: Benchmarking methods for measuring biosynthetic gene cluster similarity and determination of gene cluster families
Source: Bioinformatics. 2025 Nov 21;41(12):btaf636. doi: 10.1093/bioinformatics/btaf636 (PMC12701797; doi:10.1093/bioinformatics/btaf636)
Supplement: btaf636_Supplementary_Data [file btaf636_supplementary_data.docx]

**Supporting Information for Benchmarking Methods for Measuring Biosynthetic Gene Cluster Similarity and Determination of Gene Cluster Families**

Abiodun S. Oyedele^1^, Allison S. Walker^1,2,3^

1. Department of Chemistry, Vanderbilt University, 1234 Stevenson Center Lane, Nashville, TN 37240, United States

2. Department of Biological Sciences, Vanderbilt University, VU Station B, Box 35-1634, Nashville, TN 37235, United States

3. Department of Pathology, Microbiology, and Immunology, 1211 Medical Center Drive, Vanderbilt University Medical Center, Nashville, TN 37232, United States

address correspondence to: Allison Walker (allison.s.walker@vanderbilt.edu)

**Table S1. Butina clustering of natural products metrics.**

| Cutoff | Silhouette | Number of clusters |
| --- | --- | --- |
| 0.2 | 0.29 | 1045 |
| 0.3 | 0.29 | 920 |
| 0.4 | 0.24 | 762 |
| 0.5 | 0.19 | 582 |

**Table S2. Primary and secondary BGCs for lsaBGC run.**

| **Primary BGCs** | **Secondary BGCs** |
| --- | --- |
| BGC0001582 | BGC0001492 |
| BGC0000668 | BGC0001694 |
| BGC0000075 | BGC0001017 |
| BGC0002355 | BGC0000280 |
| BGC0001796 | BGC0002529 |
| BGC0001193 | BGC0001138 |
| BGC0001073 | BGC0002458 |
| BGC0000394 | BGC0002536 |
| BGC0000124 | BGC0001415 |
| BGC0000448 | BGC0001126 |
| BGC0001299 | BGC0001501 |
| BGC0001523 | BGC0002512 |
| BGC0000001 | BGC0001479 |
| BGC0000376 | BGC0001511 |
| BGC0000122 | BGC0002435 |
| BGC0000153 | BGC0000017 |
| BGC0001398 | BGC0000024 |
| BGC0002500 | BGC0000032 |
| BGC0000872 | BGC0002591 |
| BGC0000398 | BGC0001075 |
| BGC0000028 | BGC0001526 |
| BGC0000813 | BGC0001512 |
| BGC0000267 | BGC0002363 |
| BGC0001381 | BGC0001510 |
| BGC0000157 | BGC0002350 |
| BGC0001334 | BGC0001533 |
| BGC0000459 | BGC0000469 |
| BGC0002587 | BGC0001397 |
| BGC0000603 | BGC0002539 |
| BGC0000953 | BGC0001702 |
| BGC0001233 | BGC0000844 |
| BGC0002293 | BGC0002434 |
| BGC0000109 | BGC0002040 |
| BGC0002569 | BGC0002462 |
| BGC0000111 | BGC0001558 |
| BGC0000141 | BGC0001074 |
| BGC0000279 | BGC0000976 |
| BGC0000368 | BGC0001565 |
| BGC0002468 | BGC0001145 |
| BGC0000712 | BGC0001146 |
| BGC0001628 | BGC0000980 |
| BGC0001300 | BGC0000981 |
| BGC0001006 | BGC0000941 |
| BGC0000690 | BGC0001577 |
| BGC0000072 | BGC0002052 |
| BGC0001131 | BGC0000934 |
| BGC0000298 | BGC0002476 |
| BGC0000986 | BGC0002366 |
| BGC0001061 | BGC0000618 |
| BGC0000023 | BGC0000839 |
| BGC0001770 | BGC0002410 |
| BGC0001159 | BGC0001675 |
| BGC0002456 | BGC0001152 |
| BGC0001214 | BGC0001758 |
| BGC0002506 | BGC0002563 |
| BGC0001288 | BGC0002091 |
| BGC0000094 | BGC0002412 |
| BGC0002517 | BGC0000699 |
| BGC0001333 | BGC0000727 |
| BGC0000223 | BGC0000237 |
| BGC0000011 | BGC0001661 |
| BGC0000988 | BGC0000241 |
| BGC0002533 | BGC0001383 |
| BGC0000182 | BGC0002408 |
| BGC0000050 | BGC0001662 |
| BGC0001020 | BGC0002356 |
| BGC0001065 | BGC0001010 |
| BGC0001740 | BGC0000898 |
| BGC0000165 | BGC0000585 |
| BGC0000074 | BGC0000607 |
| BGC0000999 | BGC0002297 |
| BGC0001357 | BGC0002380 |
| BGC0001113 | BGC0001669 |
| BGC0000994 | BGC0000593 |
| BGC0000052 | BGC0000594 |
| BGC0000360 | BGC0002049 |
| BGC0001014 | BGC0000098 |
| BGC0001782 | BGC0002551 |
| BGC0000222 | BGC0002443 |
| BGC0000069 | BGC0001641 |
| BGC0000428 | BGC0001650 |
| BGC0001377 | BGC0002324 |
| BGC0000679 | BGC0002491 |
| BGC0001024 | BGC0002492 |
| BGC0001079 | BGC0001429 |
| BGC0001100 | BGC0002038 |
| BGC0001350 | BGC0002061 |
| BGC0001830 | BGC0001029 |
| BGC0000239 | BGC0001625 |
| BGC0000242 | BGC0001709 |
| BGC0000035 | BGC0001520 |
| BGC0001792 | BGC0001536 |
| BGC0000202 | BGC0001556 |
| BGC0001447 | BGC0001568 |
| BGC0002523 | BGC0001590 |
| BGC0001303 | BGC0001597 |
| BGC0001023 | BGC0001614 |
| BGC0001439 | BGC0002079 |
| BGC0001433 | BGC0000399 |
| BGC0000177 | BGC0001473 |
| BGC0000383 | BGC0001495 |
| BGC0001230 | BGC0001109 |
| BGC0001751 | BGC0002613 |
| BGC0001822 | BGC0002102 |
| BGC0001652 | BGC0002521 |
| BGC0000288 | BGC0001068 |
| BGC0001761 | BGC0001462 |
| BGC0001287 | BGC0000262 |
| BGC0001133 | BGC0000419 |
| BGC0001667 | BGC0002086 |
| BGC0000106 | BGC0001041 |
| BGC0001001 | BGC0001769 |
| BGC0001060 | BGC0001072 |
| BGC0001040 | BGC0001451 |
| BGC0000245 | BGC0002059 |
| BGC0000180 | BGC0002313 |
| BGC0000970 | BGC0001442 |
| BGC0000186 | BGC0002089 |
| BGC0001293 | BGC0001806 |
| BGC0000297 | BGC0001344 |
| BGC0001797 | BGC0002553 |
| BGC0000453 | BGC0001435 |
| BGC0000409 | BGC0001341 |
| BGC0000150 | BGC0002415 |
| BGC0000352 | BGC0002527 |
| BGC0001409 | BGC0000830 |
| BGC0000058 | BGC0001312 |
| BGC0001045 | BGC0002732 |
| BGC0000172 | BGC0002547 |
| BGC0000083 | BGC0001413 |
| BGC0000090 | BGC0000174 |
| BGC0000395 | BGC0000189 |
| BGC0001066 | BGC0000196 |
| BGC0000969 | BGC0000264 |
| BGC0001083 | BGC0001349 |
| BGC0000085 | BGC0001376 |
| BGC0000954 | BGC0001394 |
| BGC0000821 | BGC0000308 |
| BGC0000143 | BGC0000319 |
| BGC0001090 | BGC0000320 |
| BGC0000957 | BGC0000328 |
| BGC0001114 | BGC0000335 |
| BGC0000723 | BGC0001327 |
| BGC0000423 | BGC0000377 |
| BGC0001172 | BGC0000393 |
| BGC0001058 | BGC0001296 |
| BGC0000847 | BGC0000502 |
| BGC0000126 | BGC0000565 |
| BGC0000270 | BGC0000581 |
| BGC0001019 | BGC0000591 |
| BGC0000303 | BGC0000598 |
| BGC0000696 | BGC0000612 |
| BGC0000325 | BGC0001237 |
| BGC0000444 | BGC0000660 |
| BGC0000002 | BGC0000667 |
| BGC0001711 | BGC0000673 |
| BGC0000693 | BGC0000676 |
| BGC0002414 | BGC0000683 |
| BGC0002090 | BGC0001200 |
| BGC0001116 | BGC0001215 |
| BGC0000226 | BGC0000812 |
| BGC0000349 | BGC0001148 |
| BGC0000709 | BGC0000820 |
| BGC0000714 | BGC0000840 |
| BGC0001078 | BGC0000846 |
| BGC0001056 | BGC0000848 |
| BGC0000354 | BGC0000871 |
| BGC0001753 | BGC0000877 |
| BGC0000130 | BGC0000884 |
| BGC0001051 | BGC0001125 |
| BGC0000055 | BGC0000904 |
| BGC0000330 | BGC0000913 |
| BGC0000100 | BGC0000921 |
| BGC0000436 | BGC0000923 |
| BGC0000434 | BGC0000924 |
| BGC0000311 | BGC0000925 |
| BGC0001457 | BGC0000927 |
| BGC0001695 | BGC0000932 |
| BGC0001441 | BGC0000933 |
| BGC0000199 | BGC0000942 |
| BGC0000031 | BGC0000944 |
| BGC0002381 | BGC0000948 |
| BGC0001000 | BGC0000956 |
| BGC0000971 | BGC0000961 |
| BGC0001204 | BGC0000985 |
| BGC0000974 | BGC0001005 |
| BGC0002092 | BGC0001011 |
| BGC0000960 | BGC0001115 |
| BGC0000300 | BGC0001050 |
| BGC0002544 | BGC0001111 |
| BGC0001004 | BGC0001094 |
| BGC0000266 | BGC0001102 |
| BGC0002522 | BGC0001101 |
| BGC0000238 | BGC0001127 |
| BGC0001052 | BGC0001134 |
| BGC0001537 | BGC0001093 |
| BGC0000148 | BGC0001176 |
| BGC0000091 | BGC0001201 |
| BGC0000273 | BGC0001235 |
| BGC0001106 | BGC0001069 |
| BGC0000187 | BGC0001076 |
| BGC0001443 | BGC0001088 |
| BGC0000380 | BGC0001285 |
| BGC0001062 | BGC0001343 |
| BGC0000611 | BGC0001392 |
| BGC0000951 | BGC0001393 |
| BGC0000021 | BGC0001046 |
| BGC0001038 | BGC0001053 |
| BGC0000450 | BGC0001059 |
| BGC0000178 | BGC0001063 |
| BGC0001384 | BGC0001454 |
| BGC0000142 | BGC0001468 |
| BGC0000366 | BGC0001469 |
| BGC0001042 | BGC0001480 |
| BGC0000653 | BGC0001039 |
| BGC0000825 | BGC0001487 |
| BGC0000290 | BGC0001494 |
| BGC0002439 | BGC0001021 |
| BGC0000283 | BGC0001025 |
| BGC0000078 | BGC0001033 |
| BGC0001028 | BGC0001521 |
| BGC0001054 | BGC0001524 |
| BGC0002479 | BGC0000998 |
| BGC0000966 | BGC0001003 |
| BGC0001221 | BGC0001015 |
| BGC0000378 | BGC0001559 |
| BGC0001099 | BGC0001560 |
| BGC0002098 | BGC0001599 |
| BGC0000995 | BGC0001610 |
| BGC0000221 | BGC0000982 |
| BGC0002570 | BGC0000993 |
| BGC0000225 | BGC0001627 |
| BGC0000042 | BGC0001630 |
| BGC0001543 | BGC0001638 |
| BGC0001368 | BGC0001639 |
| BGC0001117 | BGC0001640 |
| BGC0000210 | BGC0000963 |
| BGC0001036 | BGC0000967 |
| BGC0000893 | BGC0000972 |
| BGC0000133 | BGC0001679 |
| BGC0000044 | BGC0001716 |
| BGC0000385 | BGC0000955 |
| BGC0000216 | BGC0001722 |
| BGC0002451 | BGC0001725 |
| BGC0000834 | BGC0001736 |
| BGC0000173 | BGC0001746 |
| BGC0001635 | BGC0001757 |
| BGC0001022 | BGC0001768 |
| BGC0000029 | BGC0001784 |
| BGC0001532 | BGC0001786 |
| BGC0000975 | BGC0001791 |
| BGC0000461 | BGC0001810 |
| BGC0000039 | BGC0001824 |
| BGC0000208 | BGC0001825 |
| BGC0000327 | BGC0002043 |
| BGC0001612 | BGC0002046 |
| BGC0000149 | BGC0002069 |
| BGC0000181 | BGC0002075 |
| BGC0002353 | BGC0002076 |
| BGC0000965 | BGC0002080 |
| BGC0001594 | BGC0002103 |
| BGC0000294 | BGC0000692 |
| BGC0000962 | BGC0000700 |
| BGC0000253 | BGC0000713 |
| BGC0002082 | BGC0000809 |
| BGC0000197 | BGC0000832 |
| BGC0001467 | BGC0000842 |
| BGC0001519 | BGC0000874 |
| BGC0000353 | BGC0000935 |
| BGC0001213 | BGC0000950 |
| BGC0000190 | BGC0000351 |
| BGC0000171 | BGC0000359 |
| BGC0000161 | BGC0000371 |
| BGC0000103 | BGC0000379 |
| BGC0000176 | BGC0000386 |
| BGC0000066 | BGC0000397 |
| BGC0000212 | BGC0000403 |
| BGC0000341 | BGC0000413 |
| BGC0001663 | BGC0000422 |
| BGC0000415 | BGC0000429 |
| BGC0000339 | BGC0000433 |
| BGC0000316 | BGC0000438 |
| BGC0000392 | BGC0000445 |
| BGC0000418 | BGC0000449 |
| BGC0000163 | BGC0000460 |
| BGC0000305 | BGC0000604 |
| BGC0000414 | BGC0000615 |
| BGC0001095 | BGC0000654 |
| BGC0000984 | BGC0002309 |
| BGC0001071 | BGC0002311 |
| BGC0000464 | BGC0002316 |
| BGC0000374 | BGC0002317 |
| BGC0000447 | BGC0000333 |
| BGC0000213 | BGC0000345 |
| BGC0000402 | BGC0002322 |
| BGC0000344 | BGC0002328 |
| BGC0000227 | BGC0002331 |
| BGC0000431 | BGC0002339 |
| BGC0000233 | BGC0002334 |
| BGC0000230 | BGC0002341 |
| BGC0000446 | BGC0002342 |
| BGC0000437 | BGC0002343 |
| BGC0000329 | BGC0002372 |
| BGC0000112 | BGC0002382 |
| BGC0000125 | BGC0002423 |
| BGC0000115 | BGC0002437 |
| BGC0000350 | BGC0000326 |
| BGC0000081 | BGC0002469 |
| BGC0000118 | BGC0002472 |
| BGC0000198 | BGC0002496 |
| BGC0000038 | BGC0000315 |
| BGC0000411 | BGC0002538 |
| BGC0000439 | BGC0000296 |
| BGC0000323 | BGC0000301 |
| BGC0000424 | BGC0002576 |
| BGC0000430 | BGC0002584 |
| BGC0000097 | BGC0000289 |
| BGC0000400 | BGC0002725 |
| BGC0000117 | BGC0002733 |
| BGC0000263 | BGC0000249 |
| BGC0000014 | BGC0000451 |
| BGC0000082 | BGC0000466 |
| BGC0000164 | BGC0000665 |
| BGC0000407 | BGC0000677 |
| BGC0000291 | BGC0000698 |
| BGC0000159 | BGC0000804 |
| BGC0000232 | BGC0000274 |
| BGC0000346 | BGC0000882 |
| BGC0000269 | BGC0000883 |
| BGC0000113 | BGC0000892 |
| BGC0000432 | BGC0000896 |
| BGC0000915 | BGC0000929 |
| BGC0000973 | BGC0001137 |
| BGC0001477 | BGC0001140 |
| BGC0000997 | BGC0001150 |
| BGC0002541 | BGC0001207 |
| BGC0002044 | BGC0001225 |
| BGC0001703 | BGC0000256 |
| BGC0002509 | BGC0000268 |
| BGC0001048 | BGC0001295 |
| BGC0000695 | BGC0001374 |
| BGC0002054 | BGC0001387 |
| BGC0000708 | BGC0001484 |
| BGC0000722 | BGC0001488 |
| BGC0001474 | BGC0001514 |
| BGC0002340 | BGC0001540 |
| BGC0002367 | BGC0000235 |
| BGC0001731 | BGC0000240 |
| BGC0001396 | BGC0001607 |
| BGC0001730 | BGC0001774 |
| BGC0000236 | BGC0001783 |
| BGC0002068 | BGC0002039 |
| BGC0001814 | BGC0000229 |
| BGC0001700 | BGC0002284 |
| BGC0001653 | BGC0002285 |
| BGC0001619 | BGC0002294 |
| BGC0001693 | BGC0002347 |
| BGC0001596 | BGC0002364 |
| BGC0001658 | BGC0002407 |
| BGC0002558 | BGC0002457 |
| BGC0002387 | BGC0002474 |
| BGC0001503 | BGC0002499 |
| BGC0001666 | BGC0002531 |
| BGC0002426 | BGC0000224 |
| BGC0001567 | BGC0002747 |
| BGC0001622 | BGC0000040 |
| BGC0001644 | BGC0000051 |
| BGC0001522 | BGC0000065 |
| BGC0000907 | BGC0000073 |
| BGC0001459 | BGC0000080 |
| BGC0000610 | BGC0000084 |
| BGC0000937 | BGC0000092 |
| BGC0001406 | BGC0000102 |
| BGC0001471 | BGC0000110 |
| BGC0000614 | BGC0000114 |
| BGC0001448 | BGC0000119 |
| BGC0001470 | BGC0000123 |
| BGC0000652 | BGC0000127 |
| BGC0001452 | BGC0000135 |
| BGC0001416 | BGC0000145 |
| BGC0001438 | BGC0000151 |
| BGC0000949 | BGC0000162 |
| BGC0000651 | BGC0000167 |
| BGC0001414 | BGC0000175 |
| BGC0000833 | BGC0000179 |
| BGC0001509 | BGC0000184 |
| BGC0001553 | BGC0000191 |
| BGC0001629 | BGC0000200 |
| BGC0001031 | BGC0000211 |
| BGC0001110 | BGC0000220 |
| BGC0001089 | BGC0001726 |
| BGC0001018 | BGC0001750 |
| BGC0001119 | BGC0001755 |
| BGC0000964 | BGC0001767 |
| BGC0001034 | BGC0001780 |
| BGC0001130 | BGC0001795 |
| BGC0000873 | BGC0001801 |
| BGC0001070 | BGC0001818 |
| BGC0001027 | BGC0001828 |
| BGC0000938 | BGC0002045 |
| BGC0001112 | BGC0002060 |
| BGC0000838 | BGC0002072 |
| BGC0002418 | BGC0002083 |
| BGC0002556 | BGC0002095 |
| BGC0002296 | BGC0002108 |
| BGC0002564 | BGC0002288 |
| BGC0002315 | BGC0002308 |
| BGC0002548 | BGC0002329 |
| BGC0002376 | BGC0002349 |
| BGC0002453 | BGC0002357 |
| BGC0002287 | BGC0002361 |
| BGC0002078 | BGC0002370 |
| BGC0002482 | BGC0002377 |
| BGC0001564 | BGC0002383 |
| BGC0001772 | BGC0002409 |
| BGC0002470 | BGC0002416 |
| BGC0001569 | BGC0002420 |
| BGC0001623 | BGC0002431 |
| BGC0001819 | BGC0002440 |
| BGC0000020 | BGC0002454 |
| BGC0001649 | BGC0002466 |
| BGC0001611 | BGC0002478 |
| BGC0001064 | BGC0002497 |
| BGC0001566 | BGC0002504 |
| BGC0001719 | BGC0002520 |
| BGC0001807 | BGC0002532 |
| BGC0001760 | BGC0002542 |
| BGC0001764 | BGC0002552 |
| BGC0001752 | BGC0002565 |
| BGC0001813 | BGC0002581 |
| BGC0001790 | |
| BGC0001646 | |
| BGC0002432 | |
| BGC0001773 | |
| BGC0001800 | |
| BGC0001823 | |
| BGC0001608 | |
| BGC0001593 | |
| BGC0001574 | |
| BGC0001091 | |
| BGC0001105 | |
| BGC0000443 | |
| BGC0002071 | |
| BGC0001332 | |
| BGC0002050 | |
| BGC0002081 | |
| BGC0002369 | |
| BGC0002460 | |
| BGC0002374 | |
| BGC0002385 | |
| BGC0002333 | |
| BGC0002085 | |
| BGC0002526 | |
| BGC0002540 | |
| BGC0002384 | |
| BGC0002419 | |
| BGC0002417 | |
| BGC0002455 | |
| BGC0002291 | |
| BGC0002048 | |
| BGC0002484 | |
| BGC0002545 | |
| BGC0002446 | |
| BGC0002109 | |
| BGC0002314 | |
| BGC0002327 | |
| BGC0002338 | |
| BGC0002379 | |
| BGC0002413 | |
| BGC0002425 | |
| BGC0002430 | |
| BGC0002433 | |
| BGC0002572 | |
| BGC0002362 | |
| BGC0002117 | |
| BGC0002505 | |
| BGC0002096 | |
| BGC0002107 | |
| BGC0002477 | |
| BGC0002351 | |
| BGC0002373 | |
| BGC0002411 | |
| BGC0002360 | |
| BGC0002358 | |
| BGC0002378 | |
| BGC0002042 | |
| BGC0002359 | |
| BGC0002070 | |
| BGC0002503 | |
| BGC0002498 | |

**
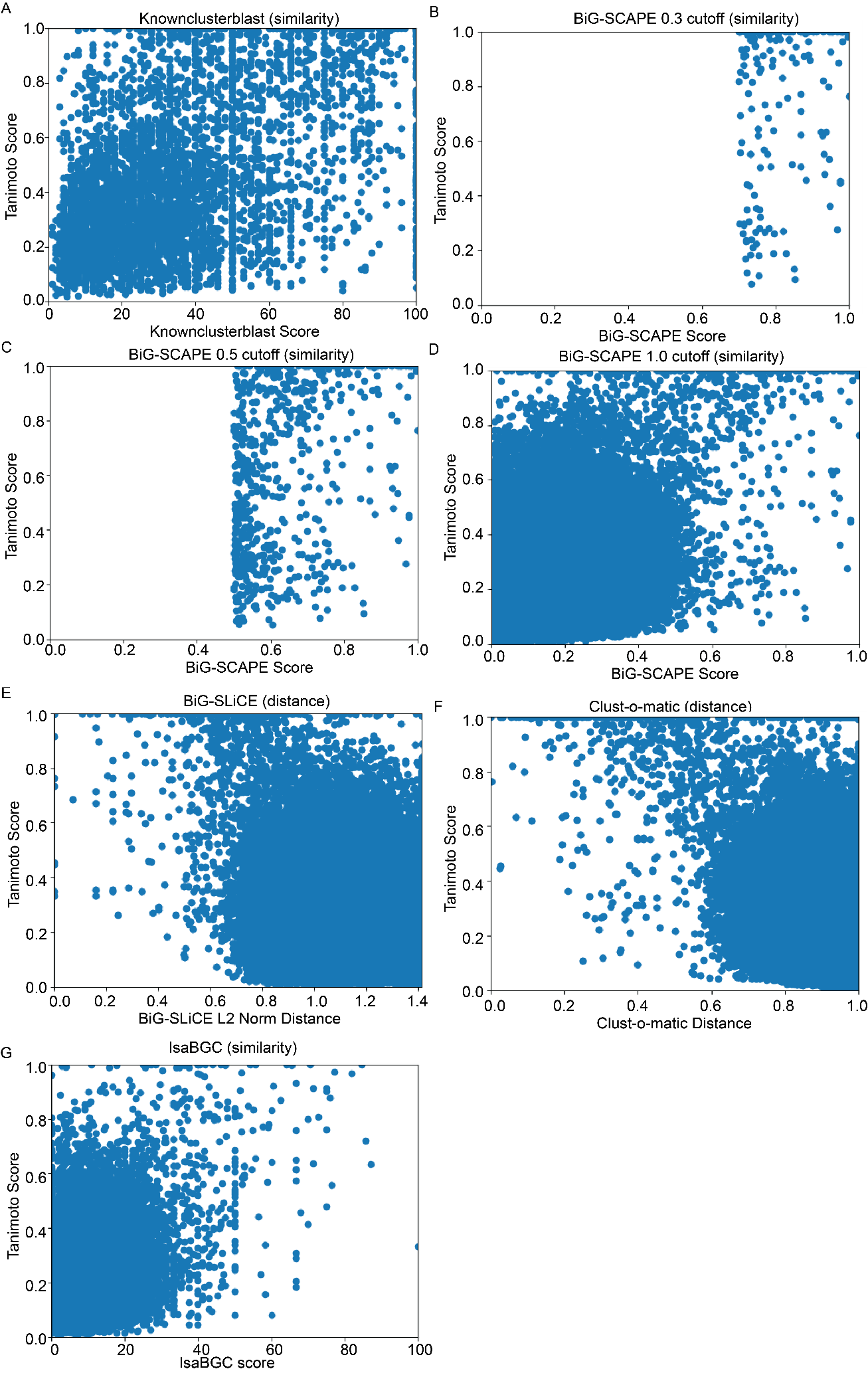
**

**Figure S1. Distance or Similarity vs Tanimoto Score.** Plots show the distance or similarity metric, as indicated by the Y axis label vs Tanimoto Score for A) knownclusterblast B) BiG-SCAPE with 0.3 similarity cutoff C)BiG-SCAPE with 0.5 similarity cutoff D) BiG-SCAPE with a 1.0 similarity cutoff E) BiG-SLiCE F) cust-o-matic G) lsaBGC.

**Table S3. BGCs Similarity Lowest Differences (High Tanimoto, Low knownclusterblast)**

In cases where the kownclusterblast is available for both directions of comparison and these values are different, a range is reported. Despite the low gene similarity, these BGCs may still produce natural products with similar structures due to the following rationales:

| **BGCs Pair** | **Score Difference** | **knownclusterblast Similarity (%)** | **Structure** | **Rationales** |
| --- | --- | --- | --- | --- |
| BGC0000612 & BGC0000607 | -0.911 | 8-28 | Thiocillin I    Micrococcin P1   | -The conserved core genes in modular biosynthesis likely produce structurally related natural products.  -Thiocillin I biosynthesis involves additional tailoring steps, including thiazole formation.  -Comparing the biosynthetic gene clusters, strain 612 contains more genes than strain 607. (Clinker)  -Gene order: The arrangement of biosynthetic genes differs between the two clusters. |
| BGC0000375 & BGC0000727 | -0.900 | 10 | Indigoidine    Indigoidine   | -Both have different export mechanisms  -Different transporter genes present.  -Regulatory elements: 727 has additional regulatory genes and accessory genes.  Cluster size: 727 cluster is larger overall.  - Gene organization: 375 cluster is more compact. |
| BGC0001109 & BGC0002068 | -0.879 | 12 | Pederin    Labrenzin   | -Conserved core biosynthetic genes.  -Different tailoring enzymes for final product modifications.  -1109 cluster includes genes for oxidative cleavage.  -2068 cluster has unique genes for pyran formation.(Kacar, et al., 2019)  -1109 cluster is split across two loci(Miller, et al., 2017), while 2068's is contiguous (as a single, contiguous genomic locus)(Kacar, et al., 2024; Kacar, et al., 2019). |
| BGC0000277 & BGC0000268 | -0.845 | 5 | Urdamycin    Sch-47554    Sch-47555   | The MIBiG entry for urdamycin BGC0000277 is truncated and missing the core PKS genes that should provide similarity to the related natural product |
| BGC0002078 & BGC0002079 | -0.840 | 57 | Omnipeptin    Omnipeptin   | -antiSMASH truncates query BGC leading to a lower KnownClusterblast score. Clinker on the full MIBiG clusters show that they are very similar with all genes having homlologs. |
| BGC0002409 & BGC0002410 | -0.840 | 16 | frankobactin **A1**-A3, B1-B3, C1    frankobactin **A1**-A3, B1-B3, C1   | -Cluster is reported as a bipartite cluster in different locations in the original paper but included as two different entries in the MIBiG database(Mohr, et al., 2021) |
| BGC000277 & BGC000229 | -0.837 | 8 | Urdamycin    Grincamycin   | The MIBiG entry for urdamycin BGC0000277 is truncated and missing the core PKS genes that should provide similarity to the related natural product |
| BGC0000277 & BGC0001769 | -0.831 | 7 | Urdamycin    Saquayamycin A   | The MIBiG entry for urdamycin BGC0000277 is truncated and missing the core PKS genes that should provide similarity to the related natural product. |
| BGC0000277 & BGC0001384 | -0.820 | 8 | Urdamycin    saprolmycin E | The MIBiG entry for urdamycin BGC0000277 is truncated and missing the core PKS genes that should provide similarity to the related natural product. |

**Figure S2. Clinker comparison of BGCs with high Tanimoto and low KnownClusterBlast scores.**
BGC0000612 & BGC0000607


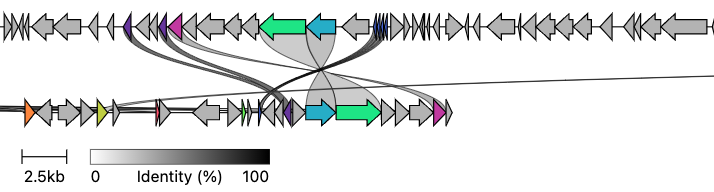


BGC0000375 & BGC0000727


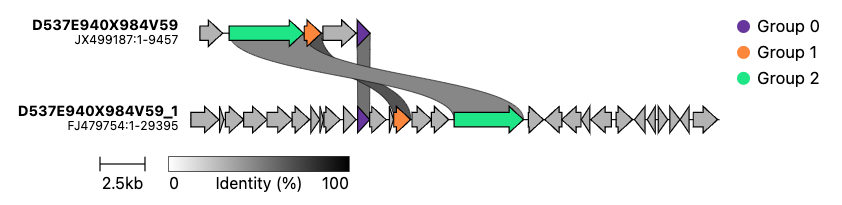


BGC0001109 & BGC0002068


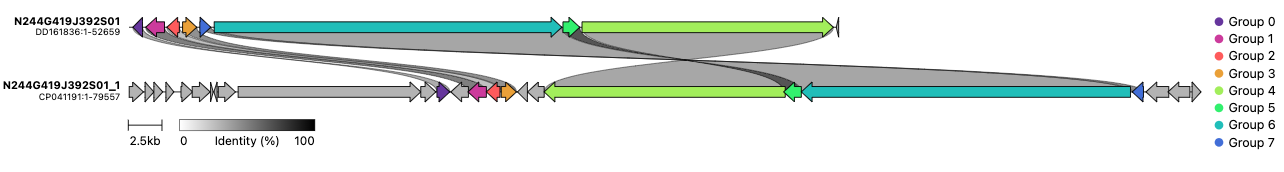


BGC0000277 & BGC0000268


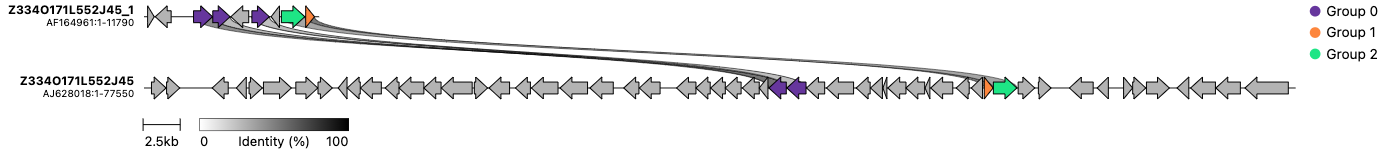


BGC0002078 & BGC0002079


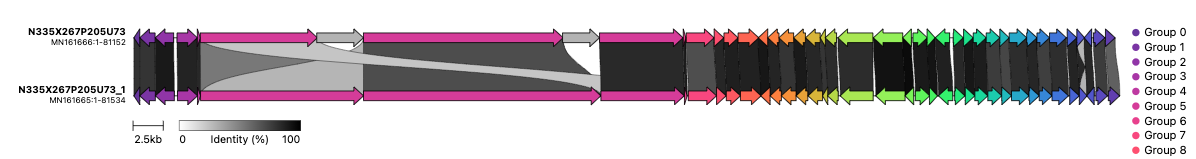


BGC0002409 & BGC0002410


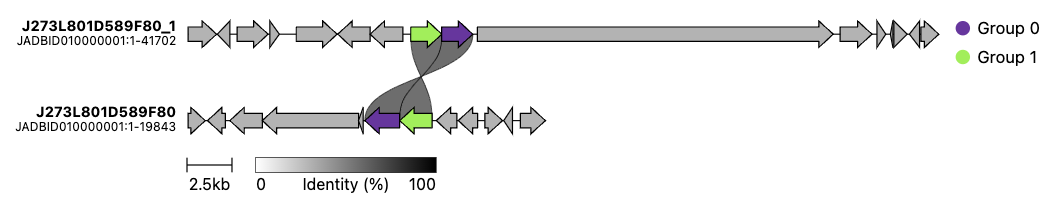


BGC0000277 & BGC0000229


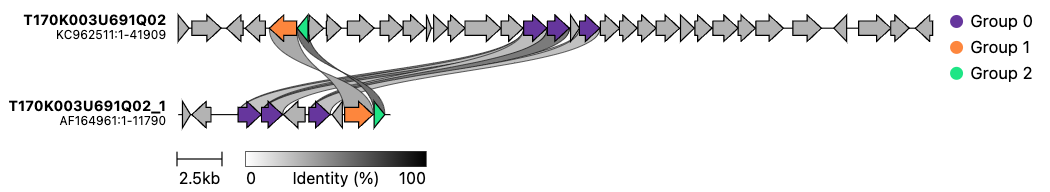


BGC0000277 & BGC0001769


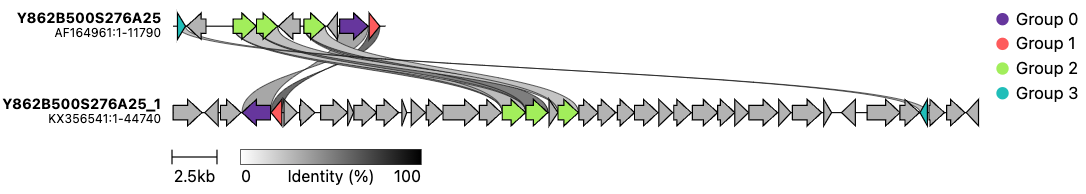


BGC0001384 & BGC0000277
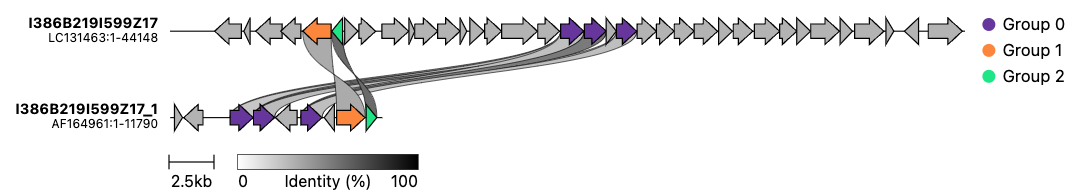


**Figure S3. knownclusterblast plots for BGCs with high Tanimoto score and low knownclusterblast score.**

BGC0000612 & BGC0000607
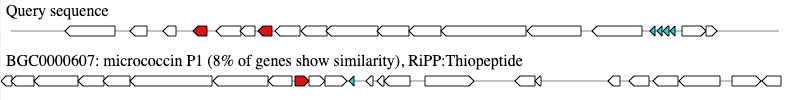


BGC0000375 & BGC0000727


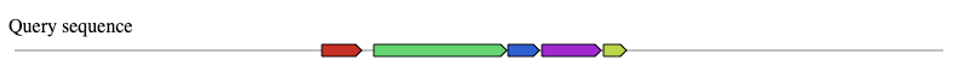


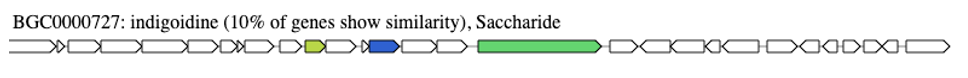


BGC0001109 & BGC0002068


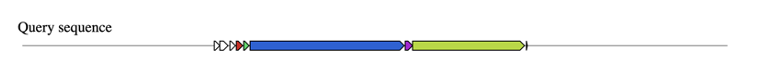


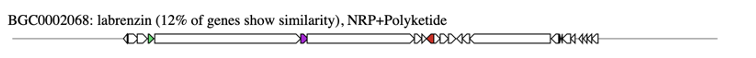


BGC0000277 & BGC0000268


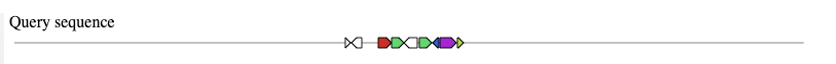


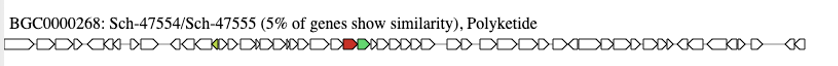


BGC0002078 & BGC0002079


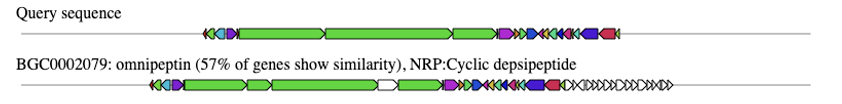


BGC0002409 & BGC0002410


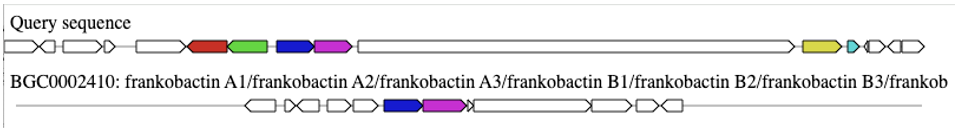


BGC0000277 & BGC0000229


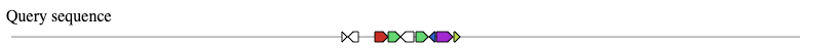


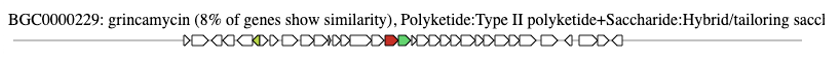


BGC0000277 & BGC0001769


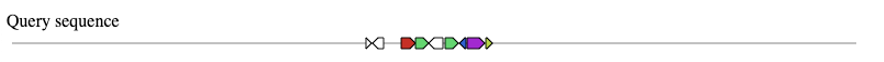


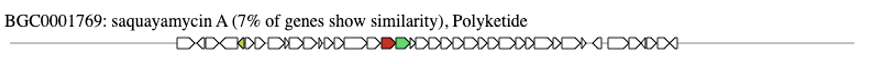


BGC0002079 & BGC0002078


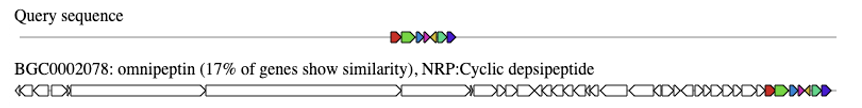


BGC0000277 & BGC0001384


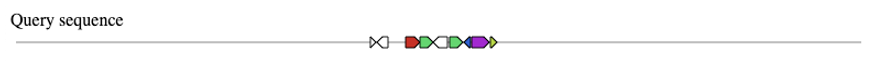

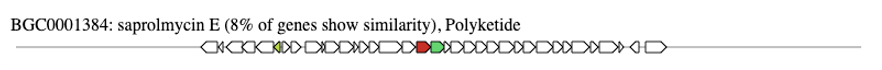


**Table S4: BGCs Similarity Highest Differences (Low Tanimoto, high knownclusterblast)**

The knownclusterblast likely gave a high similarity score for the pair of BGCs due to the following rationales:

| **BGCs Pair** | **Score Difference** | **knownclusterblast Similarity (%)** | **Structure** | **Rationales** |
| --- | --- | --- | --- | --- |
| BGC0001650 & BGC0001128 | 0.949 | 100 | Le-pyrrolopyrazines  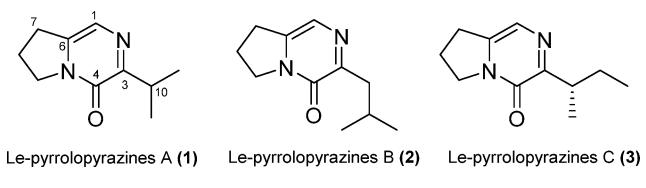  Gamexpeptide C   | Both BGCs involve NRPS genes which meet the knownclusterblast sequence similarity threshold to be counted as similar and gamexpeptide C does not have other genes in the cluster. However closer inspection reveals that the NRPS genes are not that similar – they have different number of modules that do not meet the default Clinker threshold for homology. This is a limitation of the knownclusterblast methodology |
| BGC0001816 & BGC0002193 | 0.910 | 100 | Valactamide A    Phenguignardic acid   | - The NRPS modules in the Valactamide A biosynthetic cluster exhibit functional domains similar to those found in the Phenguignardic acid cluster, particularly in the initial adenylation and thiolation domains.  -Specific Gene Arrangement and Modular Composition:  The gene cluster for Valactamide A has many more genes than the Phenguignardic acid cluster that do not contribute to the KnownClusterBlast score. |
| BGC0001703 & BGC0002480 | 0.909 | 100 | Nocuolin A    Chlorosphaerolactylate A, B, C, **D**   | - 1703 is the larger BGC and all of the genes in 2480 have a homolog in 1703. The additional genes in 1703 likely explain the structural differences in the product.  - 1703 contains 2 genes (nocN and nocO) with homology to halogenases from 2480 but no halogenated analogs of Noculin A have been discovered – this could mean these genes are inactivated or not expressed or that the halogenated analog was produced but missed in the original study |
| BGC0001433 & BGC0001296 | 0.905 | 100 | Argimycin **PI**, PII, PIV, PV, PVI, PIX  ,  Argimycin PIV    Nigrifactin    Streptazone E   | - Both Argimycin and Streptazone E involve PKS systems in their biosynthesis, indicating common use of polyketide assembly line mechanisms.  - Nigrifactin is an intermediate in the pathway for both BGCs and is observed in extracts from cultures of both producers,(Ohno, et al., 2015; Ye, et al., 2018; Ye, et al., 2017) it is the only product from 1433 listed in NPAtlas, while it is not listed as a product of 1296 in either MIBiG or NPAtlas leading to a Tanimoto score that is lower than it should be |
| BGC0002295 & BGC0000375 | 0.891 | 100 | Minimycin    Indigoidine   | - Both Minimycin and Indigoidine involve similar NRPS biosynthetic pathways.  - BGC 2295 is reported to produce both minimycin and indigoidine(Kong, et al., 2019) but has only minimycin as a product in NPAtlas  - It is possible that 375 also produces both products but that minimycin was missed as a product of the BGC or that regulation of product production differs between the two clusters |
| BGC0001048 & BGC0001128 | 0.876 | 100 | Tallysomycin **A**, B    Gamexpeptide C   | - Both Tallysomycin A, B and Gamexpeptide C involve NRPS systems for peptide assembly.  - The Tallysomycin biosynthetic gene cluster contains a unique set of polyketide synthase genes adjacent to the NRPS modules, unlike the Gamexpeptide biosynthetic cluster which strictly involves NRPS genes.  - the NRPS gene is the only gene in the Gamexpeptide cluster meets the similarity threshold for homology to a gene in the tallysomycin cluster despite not having the same number of modules and not meeting the default threshold for homology in clinker |
| BGC0001405 & BGC0002608 | 0.869 | 100 | Chaetoviridin E    11-epichaetomugilin A  Ochratoxin A   | The MIBiG entry for 2608 only has a single gene, a halogenase, which has homology to a gene in 1405, leading to the high knownclusterblast score, however the original publication reports that there are 5 ORFs in the region they identified as the ochratoxin BGC, including a polyketide synthase the MIBiG entry lacks. Therefore, the MIBiG entry is likely either truncated or contains genes that were not annotated. |
| BGC0002426 & BGC0001512 | 0.866 | 100 | Svetamycin **A,** H, Deschlorosvetamycin A    BE-24566B   | The BGC annotated as svetamycin in MIBiG seems to actually the BGC for ABXs and borregomycins, reported in the same paper as the BGC for svetamycin. The ABXs are more similar to BE-34566B(Morshed, et al., 2021). |
| BGC0000098 & BGC0002192 | 0.860 | 100 | Monacolin K    FR901512   | -Significant conserved core region: The PKS modules in both Monacolin K and FR901512 biosynthetic pathways exhibit conserved acyltransferase, ketosynthase, and acyl carrier protein domains, which are crucial for polyketide chain elongation  - The 2192 BGC is likely truncated as there is only a single gene when the original paper reports six(Itoh, et al., 2018) and the homology of this gene to the other BGC leads to an inflated knownclusterblast score. |

**Figure S4 – BGCs with high knownclusterblast scores and low Tanimoto scores analyzed with clinker.**

BGC0001650 & BGC0001128


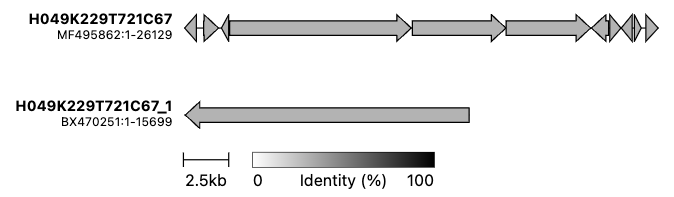


BGC0001816 & BGC0002193


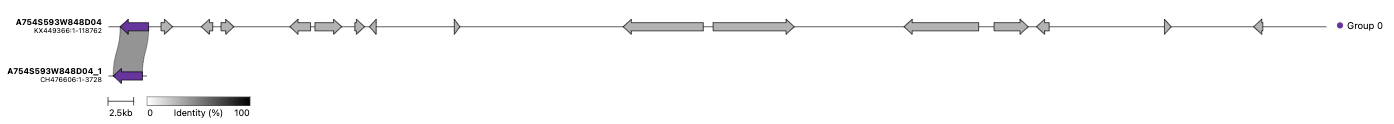


BGC0001703 & BGC0002480


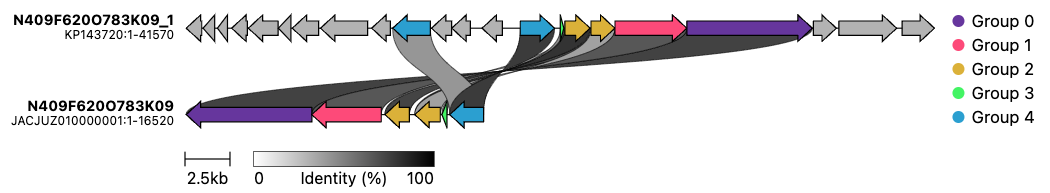


BGC0001433 & BGC0001296


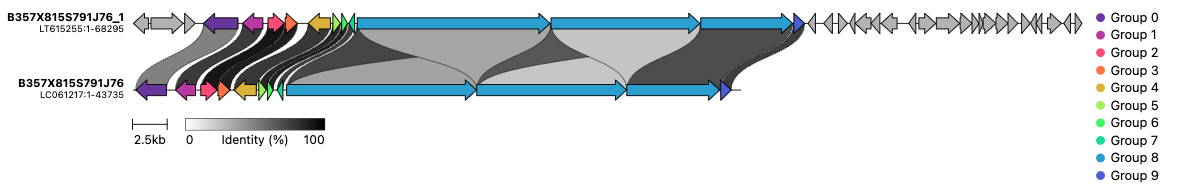


BGC0000375 & BGC0002295


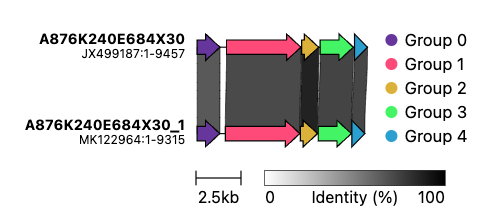


BGC0001048 & BGC0001128


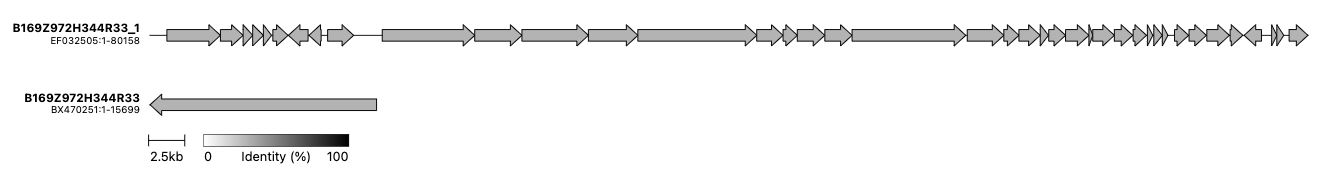


BGC0002608 & BGC0001405


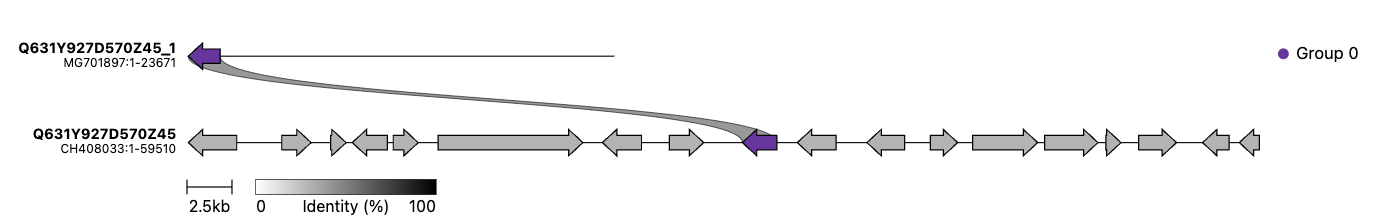


BGC0001512 & BGC0002426


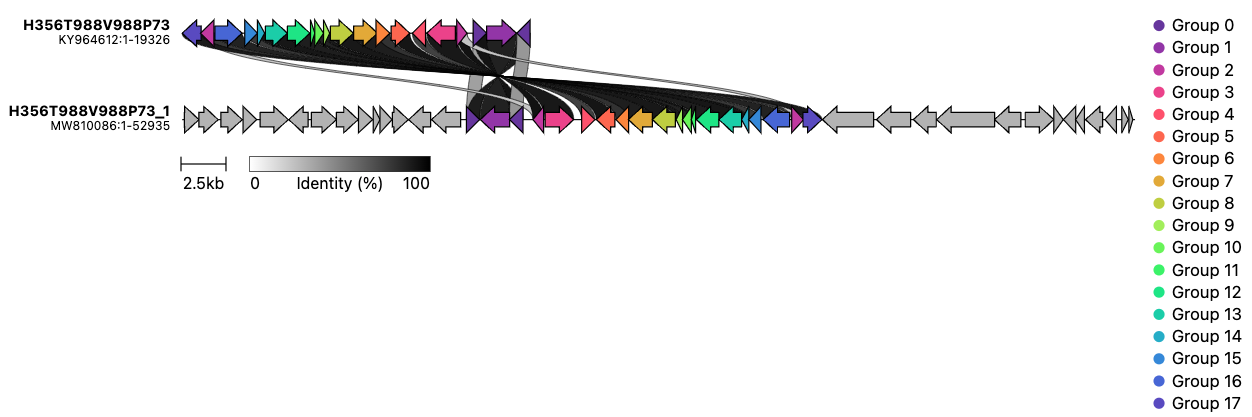


BGC0000098 & BGC0002192


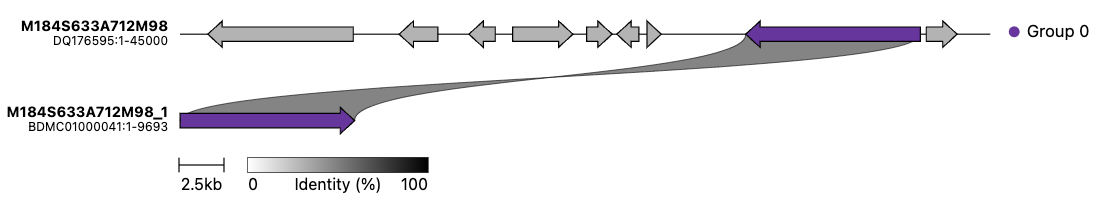


**Figure S5. knownclusterblast plots for BGCs with low Tanimoto score and high knownclusterblast score.**

BGC0001650 & BGC0001128


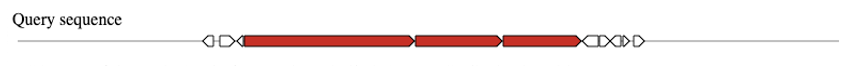


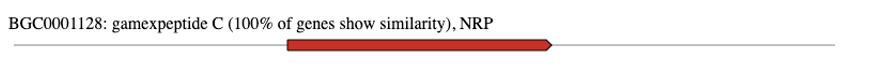


BGC0001816 & BGC0002193


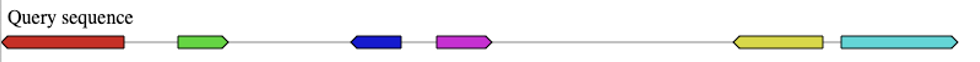


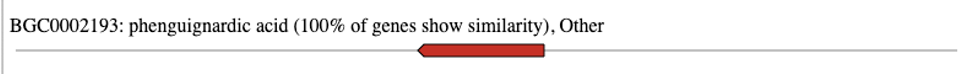


BGC0001703 & BGC0002480


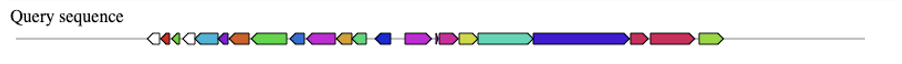

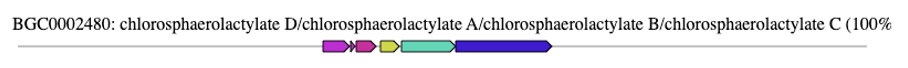


BGC0001433 & BGC0001296


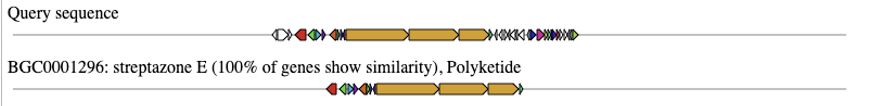


BGC0002295 & BGC0000375


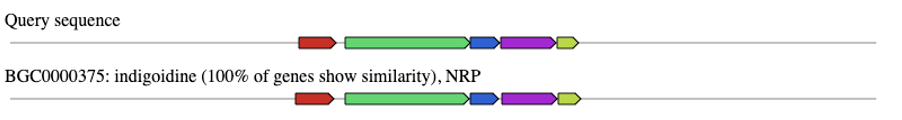


BGC0000375 & BGC0002295


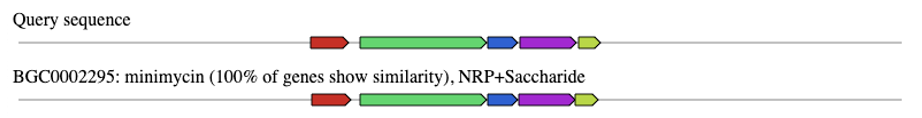


BGC0001048 & BGC0001128


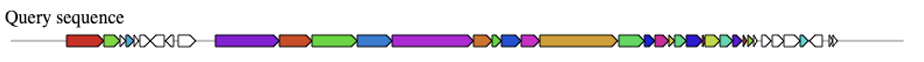

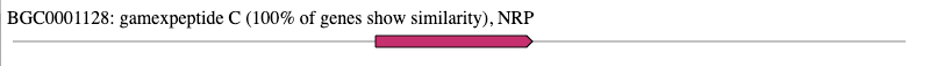


BGC0001405 & BGC0002608


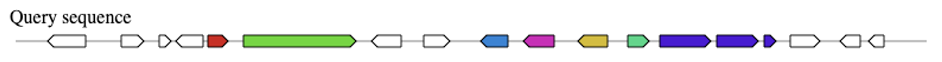

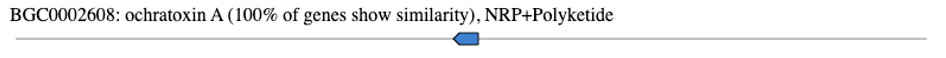


BGC0002426 & BGC0001512


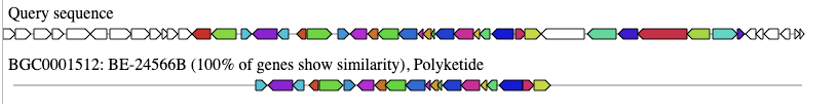


BGC0000098 & BGC0002192


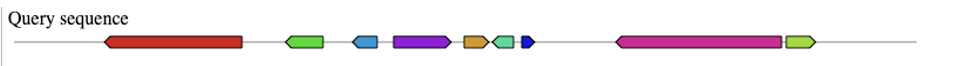

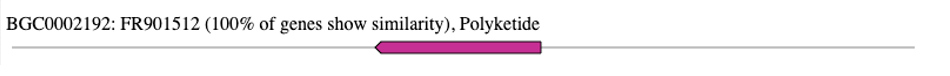


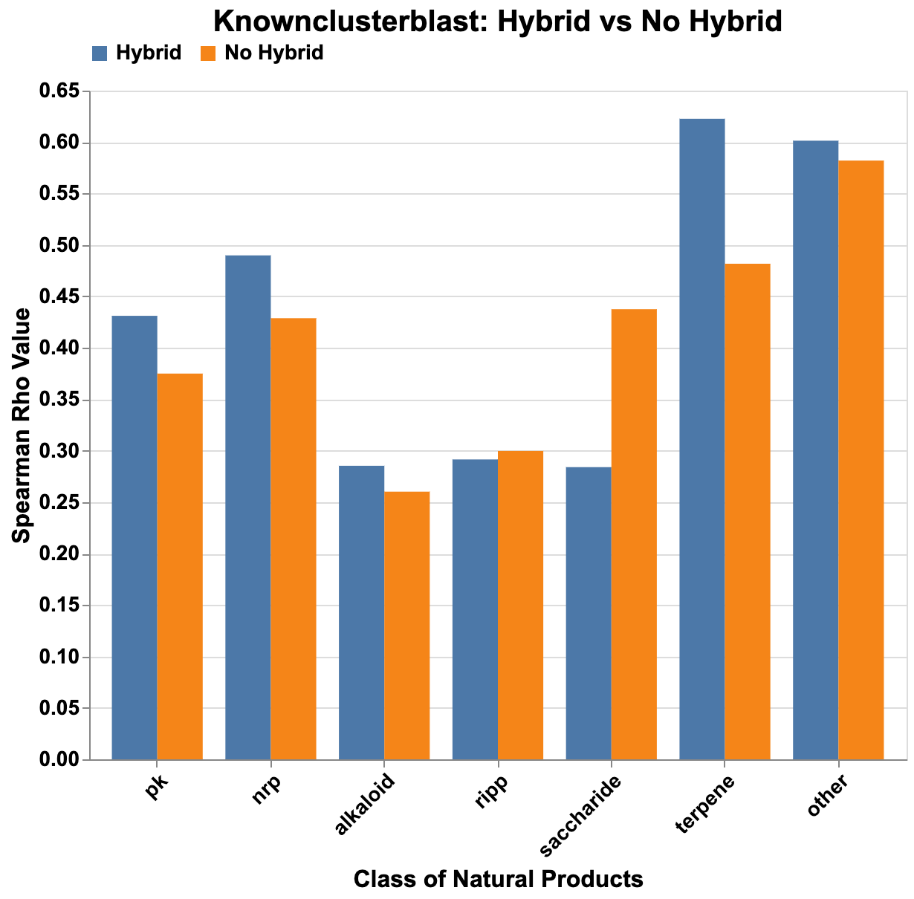


**Figure S6. Comparison of knownclusterblast performance on different natural product classes.**


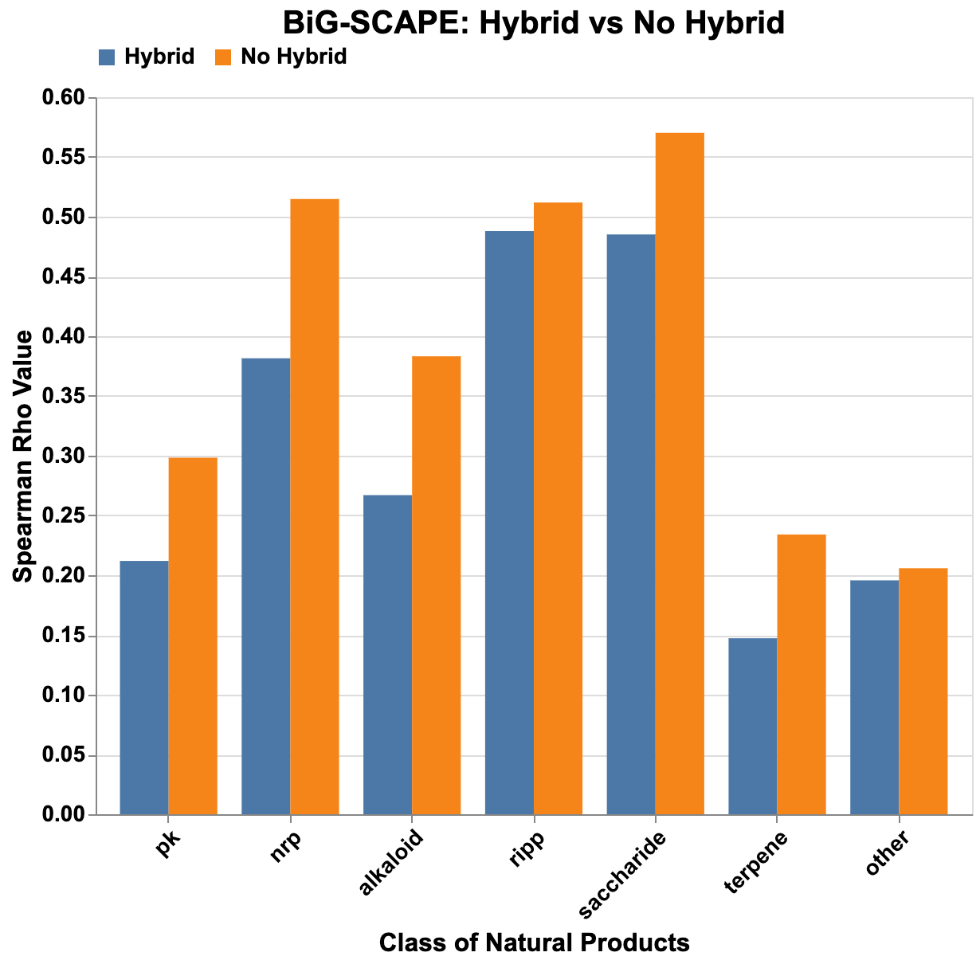


**Figure S7. Comparison of BiG-SCAPE performance on different natural product classes.**


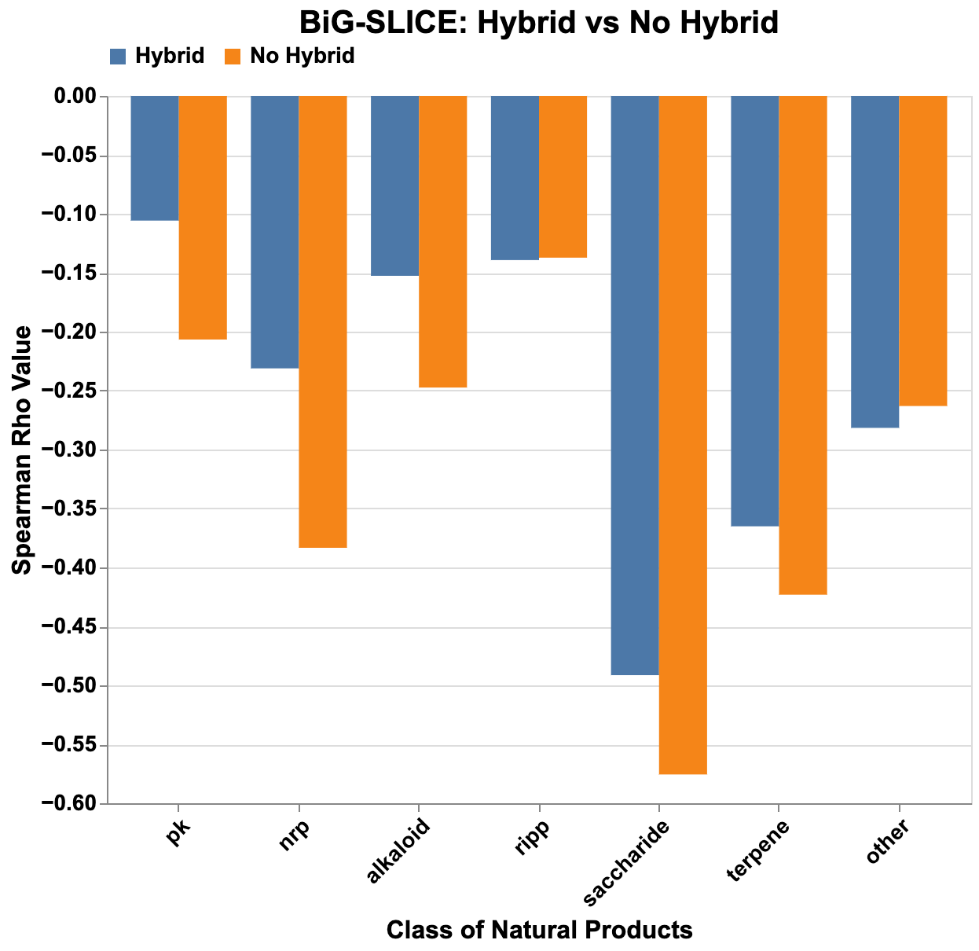


**Figure S8. Comparison of BiG-SLiCE performance on different natural product classes.**


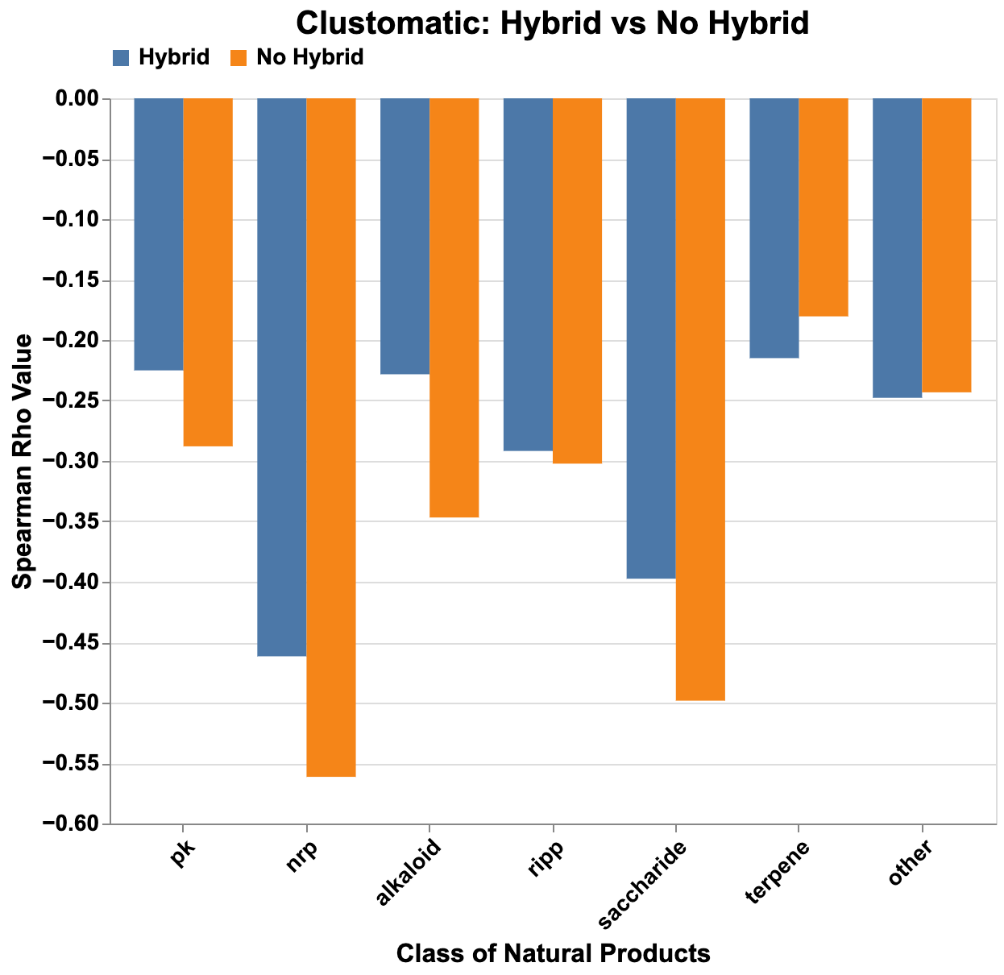


**Figure S9. Comparison of clust-o-matic performance on different natural product classes.**


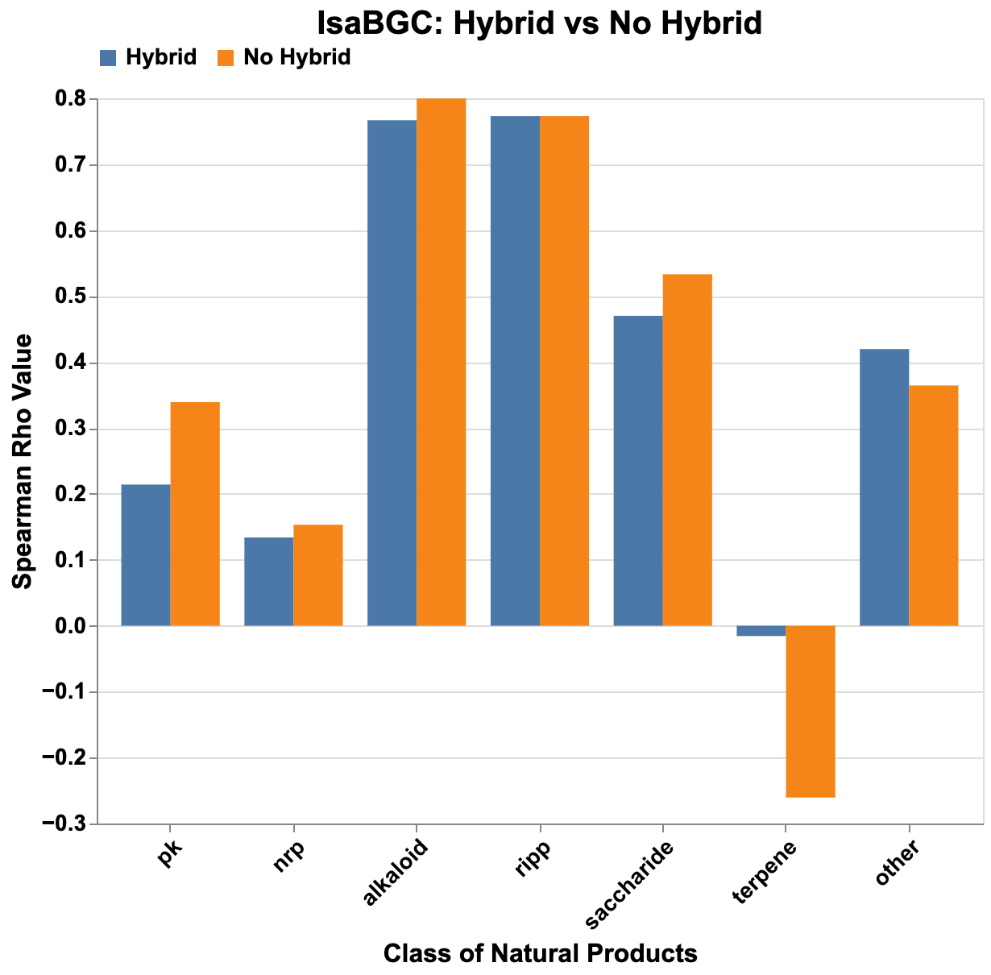


**Figure S10. Comparison of lsaBGC performance on different natural product classes.**

**Table S5. Spearman’s correlation for different natural product classes and distance measurement methods for BGCs used in lsaBGC method.** The best score for a class is bold and the best score for a method is underlined. The upper part of the table includes hybrid clusters (those with more than one biosynthetic class) while the bottom part of the table excludes them. Correlations are rounded to the nearest hundredth, but further decimals were used to break ties when determining the best score.

| Including Hybrid Clusters | | | | | | | |
| --- | --- | --- | --- | --- | --- | --- | --- |
|  | PK | NRP | alkaloid | RiPP | Saccharide | Terpene | Other |
| Knownclusterblast | **0.44** | **0.51** | 0.17 | 0.38 | 0.29 | **0.98** | **0.66** |
| BiG-SCAPE 1.0 cutoff | 0.31 | 0.43 | **0.38** | **0.62** | **0.50** | 0.24 | 0.17 |
| BiG-SLiCE | -0.18 | -0.22 | -0.25 | -0.37 | -0.51 | -0.43 | -0.37 |
| Clust-o-matic | -0.33 | -0.51 | -0.42 | -0.44 | -0.44 | -0.24 | -0.27 |
| No Hybrid Clusters | | | | | | | |
| Knownclusterblast | 0.40 | 0.52 | 0.26 | 0.38 | 0.48 | ND | **0.74** |
| BiG-SCAPE 1.0 cutoff | 0.43 | **0.54** | **0.72** | **0.64** | **0.62** | 0.40 | 0.16 |
| BiG-SLiCE | -0.31 | -0.37 | -0.55 | -0.37 | -0.60 | **-0.44** | -0.36 |
| Clust-o-matic | **-0.42** | -0.57 | -0.68 | -0.47 | -0.50 | -0.15 | -0.27 |

**Table S6. Spearman’s correlation for different natural product classes and distance measurement methods for pairs of BGCs lsaBGC recognizes.** The best score for a class is bold and the best score for a method is underlined. The upper part of the table includes hybrid clusters (those with more than one biosynthetic class) while the bottom part of the table excludes them. Correlations are rounded to the nearest hundredth, but further decimals were used to break ties when determining the best score. ND indicates that there were not enough pairs to calculate the correlation coefficient.

| Including Hybrid Clusters | | | | | | | |
| --- | --- | --- | --- | --- | --- | --- | --- |
|  | PK | NRP | alkaloid | RiPP | Saccharide | Terpene | Other |
| Knownclusterblast | **0.39** | **0.45** | 0.72 | 0.50 | 0.17 | **0.99** | **0.5** |
| BiG-SCAPE 1.0 cutoff | 0.34 | 0.44 | 0.82 | 0.80 | 0.44 | -0.66 | 0.19 |
| BiG-SLiCE | -0.20 | -0.24 | -0.70 | **-0.90** | **-0.57** | -0.05 | -0.57 |
| Clust-o-matic | -0.34 | -0.55 | **-0.87** | -0.90 | -0.52 | -0.02 | -0.32 |
| No Hybrid Clusters | | | | | | | |
| Knownclusterblast | 0.37 | 0.54 | 0.72 | 0.5 | **-0.67** | ND | ND |
| BiG-SCAPE 1.0 cutoff | **0.46** | 0.57 | **0.90** | 0.80 | 0.52 | ND | 0.17 |
| BiG-SLiCE | -0.35 | -0.44 | -0.87 | **-0.90** | -0.51 | 0.42 | -0.58 |
| Clust-o-matic | -0.44 | **-0.64** | -0.90 | -0.90 | -0.57 | 0.47 | -0.37 |


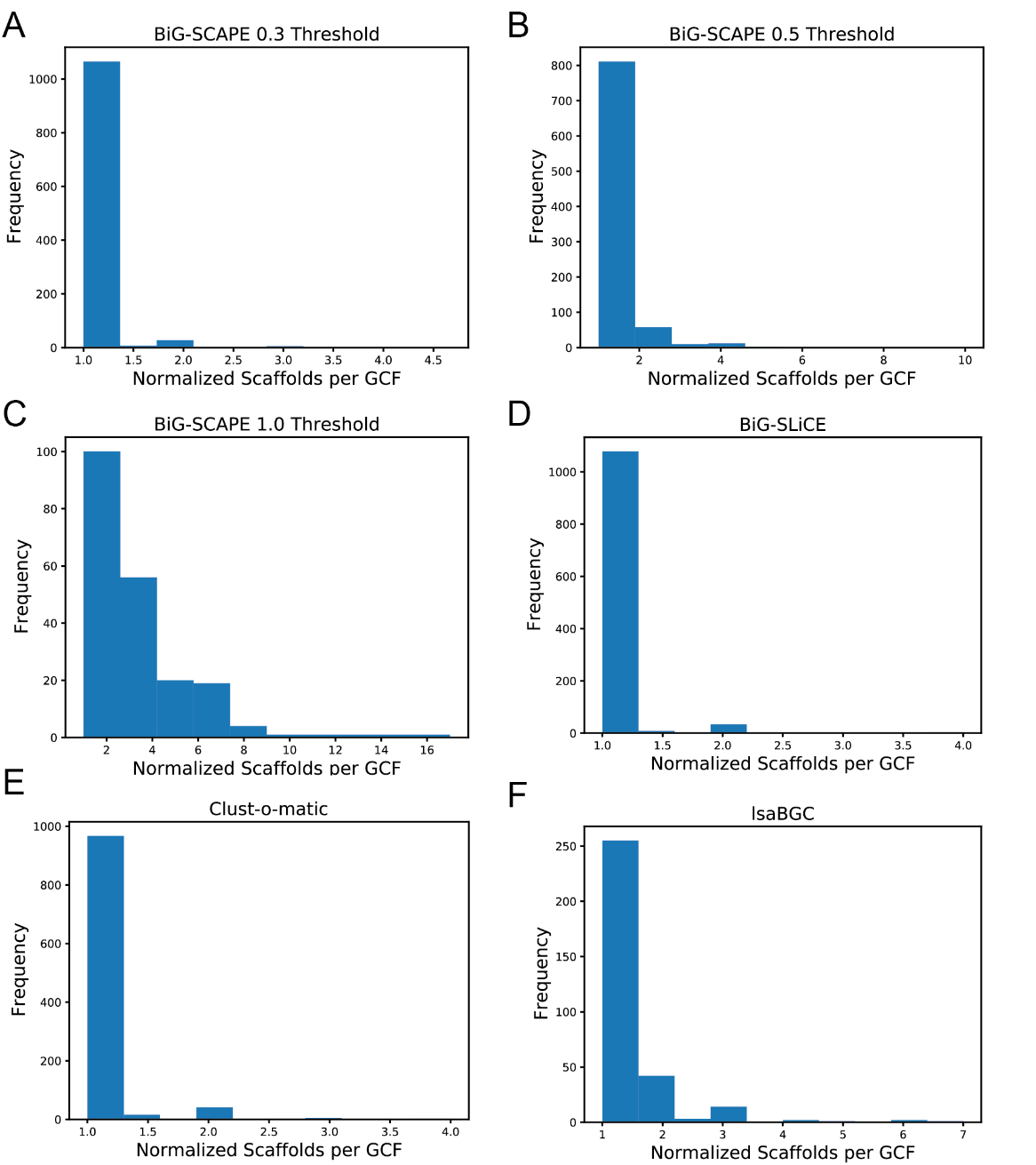


**Figure S11. Histograms of normalized scaffolds per GCF.** A) BiG-SCAPE with 0.3 cutoff B) BiG-SCAPE with 0.5 cutoff C) Big-SCAPE with 1.0 cutoff D) BiG-SLICE E) clust-o-matic F) lsaBGC


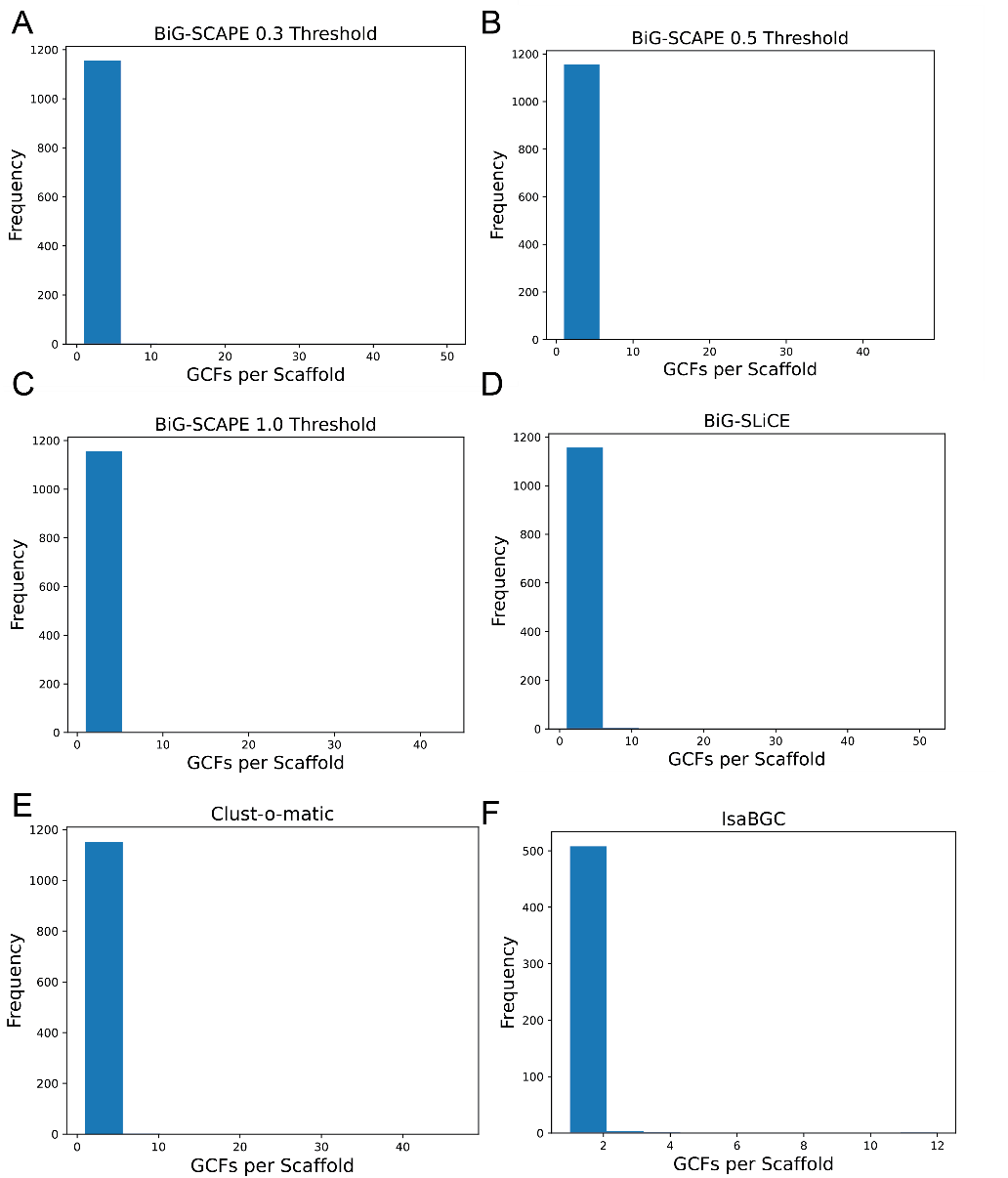


**Figure S12. Histograms of GCFs per scaffold.** A) BiG-SCAPE with 0.3 cutoff B) BiG-SCAPE with 0.5 cutoff C) Big-SCAPE with 1.0 cutoff D) BiG-SLICE E) clust-o-matic F) lsaBGC

**Table S7. Correlation for all BGCs with updated dataset**

| **Method** | **Spearman’s rho** |
| --- | --- |
| **Knownclusterblast (similarity)** | **0.48** |
| BiG-SCAPE 0.3 cutoff (similarity) | 0.28 |
| BiG-SCAPE 0.5 cutoff (similarity) | 0.42 |
| BiG-SCAPE 1 cutoff (similarity) | 0.26 |
| BiG-SLICE (distance) | -0.19 |
| Clust-o-matic 0.5 cutoff (distance) | -0.28 |
| lsaBGC (similarity) | 0.19 |

**Table S8.** **Spearman’s correlation for different natural product classes and distance measurement methods with updated dataset.** The best score for a class is bolded and the best score for a method is underlined. Hybrids are clusters that are classified by MiBIG as having multiple biosynthetic classes. The hybrid clusters in the upper part of the table are classified as a biosynthetic class if that class is listed among the biosynthetic classes on MiBIG. The no hybrid cluster category excludes all clusters with more than one biosynthetic class on MiBIG. Correlations are rounded to the nearest hundredth, but further decimals were used to break ties when determining best score if necessary.

| Hybrid Clusters | | | | | | | |
| --- | --- | --- | --- | --- | --- | --- | --- |
|  | PK | NRP | alkaloid | RiPP | Saccharide | Terpene | Other |
| Knownclusterblast | **0.44** | **0.49** | 0.25 | 0.29 | 0.25 | **0.62** | **0.60** |
| BiG-SCAPE 1.0 cutoff | 0.22 | 0.38 | 0.27 | 0.49 | 0.49 | 0.15 | 0.20 |
| BiG-SLiCE | -0.11 | -0.23 | -0.15 | -0.13 | **-0.49** | -0.37 | -0.28 |
| Clust-o-matic | -0.23 | -0.46 | -0.23 | -0.29 | -0.40 | -0.21 | -0.25 |
| lsaBGC | 0.21 | 0.14 | **0.77** | **0.77** | 0.47 | -0.02 | 0.42 |
| No Hybrid Clusters | | | | | | | |
| Knownclusterblast | **0.39** | 0.43 | 0.26 | 0.30 | 0.44 | **0.48** | **0.58** |
| BiG-SCAPE 1.0 cutoff | 0.31 | 0.52 | 0.38 | 0.51 | 0.57 | 0.23 | 0.21 |
| BiG-SLiCE | -0.21 | -0.39 | -0.25 | -0.13 | **-0.58** | -0.43 | -0.26 |
| Clust-o-matic | -0.30 | **-0.57** | -0.35 | -0.30 | -0.50 | -0.18 | -0.24 |
| lsaBGC | 0.34 | 0.16 | **0.80** | **0.77** | 0.53 | -0.26 | 0.36 |

**Table S9. Silhouette clustering scores on new dataset.** Best scores are shown in bold.

|  | Silhouette – Method Score | Silhouette – Structure Tanimoto |
| --- | --- | --- |
| BiG-SCAPE 0.3 cutoff | **0.70** | **0.60** |
| BiG-SCAPE 0.5 cutoff | 0.50 | 0.37 |
| BiG-SCAPE 1.0 cutoff | 0.09 | -0.03 |
| BiG-SLiCE | 0.09 | 0.04 |
| Clust-o-matic 0.5 cutoff | 0.14 | 0.10 |
| lsaBGC | 0.01 | -0.07 |

**Table S10. Supervised clustering metrics on new dataset.** Best scores are shown in bold.

|  | Adjusted rand | Adjusted mutual information | V-measure | Fowlkes Mallow |
| --- | --- | --- | --- | --- |
| Butina threshold=0.2 | | | | |
| BiG-SCAPE 0.3 cutoff | **0.66** | **0.74** | 0.95 | **0.68** |
| BiG-SCAPE 0.5 cutoff | 0.46 | 0.57 | 0.92 | 0.52 |
| BiG-SCAPE 1.0 cutoff | 0.13 | 0.24 | 0.84 | 0.21 |
| BiG-SLiCE | 0.38 | 0.45 | 0.96 | 0.38 |
| Clust-o-matic 0.5 cutoff | 0.43 | 0.49 | **0.97** | 0.46 |
| lsaBGC | 0.19 | 0.26 | 0.94 | 0.23 |
| Butina threshold=0.3 | | | | |
| BiG-SCAPE 0.3 cutoff | **0.64** | **0.75** | 0.95 | **0.64** |
| BiG-SCAPE 0.5 cutoff | 0.49 | 0.61 | 0.92 | 0.50 |
| BiG-SCAPE 1.0 cutoff | 0.15 | 0.29 | 0.83 | 0.19 |
| BiG-SLiCE | 0.26 | 0.40 | 0.95 | 0.30 |
| Clust-o-matic 0.5 cutoff | 0.26 | 0.41 | **0.96** | 0.34 |
| lsaBGC | 0.22 | 0.29 | 0.93 | 0.23 |

**Table S11. Molecular scaffolds and GCFs on new dataset.**

| Clustering Method | Normalized Scaffold per GCF | GCFs per Scaffold |
| --- | --- | --- |
| BiG-SCAPE 0.3 cutoff | 1.46 | **1.04** |
| BiG-SCAPE 0.5 cutoff | 1.90 | 1.06 |
| BiG-SCAPE 1.0 cutoff | 3.35 | 1.14 |
| BiG-SLiCE | 1.15 | 1.19 |
| Clust-o-matic 0.5 cutoff | **1.07** | 1.19 |
| lsaBGC | 1.32 | 1.11 |

**References**

Itoh, H.*, et al.* Biosynthesis of Novel Statins by Combining Heterologous Genes from Xylaria and Aspergillus. *ACS Synth Biol* 2018;7(12):2783-2789.

Kacar, D.*, et al.* Tailoring modifications in labrenzin synthesis: a-la-carte production of pathway intermediates. *Microb Biotechnol* 2024;17(1):e14355.

Kacar, D.*, et al.* Genome of Labrenzia sp. PHM005 Reveals a Complete and Active Trans-AT PKS Gene Cluster for the Biosynthesis of Labrenzin. *Front Microbiol* 2019;10:2561.

Kong, L.*, et al.* Divergent Biosynthesis of C-Nucleoside Minimycin and Indigoidine in Bacteria. *Iscience* 2019;22:430-440.

Miller, I.J., Chevrette, M.G. and Kwan, J.C. Interpreting Microbial Biosynthesis in the Genomic Age: Biological and Practical Considerations. *Mar Drugs* 2017;15(6).

Mohr, J.F.*, et al.* Frankobactin Metallophores Produced by Nitrogen-Fixing Frankia Actinobacteria Function in Toxic Metal Sequestration. *J Nat Prod* 2021;84(4):1216-1225.

Morshed, M.T.*, et al.* Chlorinated metabolites from Streptomyces sp. highlight the role of biosynthetic mosaics and superclusters in the evolution of chemical diversity. *Org Biomol Chem* 2021;19(27):6147-6159.

Ohno, S.*, et al.* Identification and Characterization of the Streptazone E Biosynthetic Gene Cluster in Streptomyces sp. MSC090213JE08. *Chembiochem* 2015;16(16):2385-2391.

Ye, S.*, et al.* New Insights into the Biosynthesis Pathway of Polyketide Alkaloid Argimycins P in Streptomyces argillaceus. *Front Microbiol* 2018;9:252.

Ye, S.*, et al.* Identification by Genome Mining of a Type I Polyketide Gene Cluster from Streptomyces argillaceus Involved in the Biosynthesis of Pyridine and Piperidine Alkaloids Argimycins P. *Front Microbiol* 2017;8:194.
